# The effect of specific adsorption of halide ions on electrochemical CO_2_ reduction†

**DOI:** 10.1039/d2sc02689a

**Published:** 2022-06-28

**Authors:** Tenghui Yuan, Tuo Wang, Gong Zhang, Wanyu Deng, Dongfang Cheng, Hui Gao, Jing Zhao, Jia Yu, Peng Zhang, Jinlong Gong

**Affiliations:** School of Chemical Engineering and Technology, Key Laboratory for Green Chemical Technology of Ministry of Education, Tianjin University Tianjin 300072 China jlgong@tju.edu.cn; Collaborative Innovation Center for Chemical Science & Engineering Tianjin 300072 China; Haihe Laboratory of Sustainable Chemical Transformations Tianjin 300192 China; Joint School of National University of Singapore and Tianjin University, International Campus of Tianjin University Binhai New City Fuzhou 350207 China

## Abstract

In the electrochemical CO_2_ reduction reaction (CO_2_RR), halide ions could impose a significant effect on multi-carbon (C_2+_) product production for Cu-based catalysts by a combined contribution from various mechanisms. However, the nature of specific adsorption of halide ions remains elusive due to the difficulty in decoupling different effects. This paper describes a facile method to actively immobilize the morphology of Cu-based catalysts during the CO_2_RR, which makes it possible to reveal the fundamental mechanism of specific adsorption of halide ions. A stable morphology is obtained by pre-reduction in aqueous KX (X = Cl, Br, I) electrolytes followed by conducting the CO_2_RR using non-buffered and non-specifically adsorbed K_2_SO_4_ as the supporting electrolyte, by which the change of local pH and cation concentration is also maintained during the CO_2_RR. *In situ* spectroscopy revealed that the specific adsorption of halide ions enhances the adsorption of *CO intermediates, which enables a high selectivity of 84.5% for C_2+_ products in 1.0 M KI.

## Abstract 2

Reconstruction of catalyst morphology induced by halide ions over of Cu-based catalysts during CO_2_ electroreduction is suppressed using K_2_SO_4_ as supporting electrolyte. Adsorption of halide ions is an enabling factor to enhance CO_2_ electroreduction.

## Graphical abstract

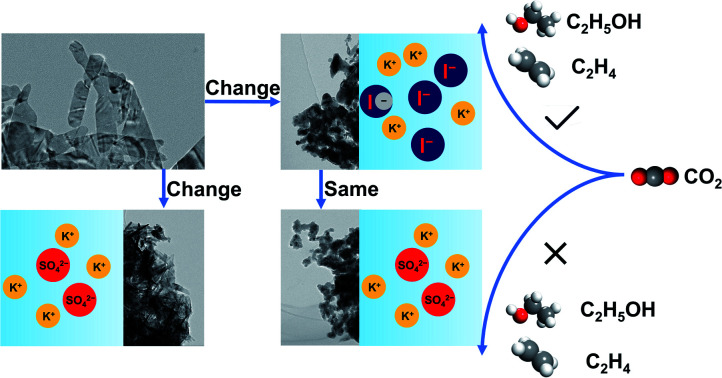

## Introduction

The electrochemical CO_2_ reduction reaction (CO_2_RR) driven by renewable electricity, such as solar and wind power, holds great potential to close the carbon cycle.^1–3^ Up to now, Cu-based materials have attracted extensive attention since they are the only transition metal-based catalysts known to catalyze the high-rate electroreduction of CO_2_ to multi-carbon (C_2+_) products (*e.g.*, C_2_H_4_ and C_2_H_5_OH).^4,5^ The composition of the aqueous electrolyte has been widely recognized as a critical factor affecting the catalytic activity and selectivity of copper.^6–13^ For cations, Hori *et al.* and Bell *et al.* ascribed the promoter effect of alkali metal cations to the change of outer Helmholtz plane potential^14^ and the interfacial electric field,^8,9^ respectively. Recently, Xu *et al.* revealed that the increase in cation concentration promotes the formation of C_2+_.^15^ For anions,^16,17^ Hori *et al.* reported that non-buffered anions (Cl^−^, ClO_4_^−^, and SO_4_^2−^) enhance the CO_2_RR selectivity towards C_2+_ while the buffered anions (HCO_3_^−^ and HPO_4_^2−^) promote the formation of H_2_ and CH_4_, and their production rates increase with the increasing concentration of buffered anions, which can be ascribed to the local pH during the CO_2_RR, because higher local pH facilitates the production of C_2+_.^18–20^ Thus, the anions and cations in aqueous electrolytes may affect the CO_2_RR through various pathways.

Among different ions in aqueous electrolytes, halide ions have attracted broad interest due to their specific adsorption on catalysts. It has been reported that the reconstruction of the catalyst surface and charge transfer induced by specific adsorption of halide ions could enhance the selectivity/activity towards C_2+_ and/or other CO_2_RR products, making it a promising approach to tune the product distribution of the CO_2_RR by optimizing the type and concentration of halide ions.^17,21–26^ It has been widely accepted that halide ions could easily induce the reconstruction of Cu,^13,21–24^ leading to the changes in the strain effect, exposed active sites, surface roughness, *etc.* Moreover, the reconstructed morphologies vary with the types of halide ions, resulting in different activity/selectivity towards the CO_2_RR, making it difficult to compare the CO_2_RR activity and selectivity in different aqueous halide containing electrolytes.^13,23,25^

In addition to inducing morphological changes, the specific adsorption of halide ions on Cu is also reported to interact with reaction intermediate species directly and affect the product distribution. A lot of insightful understandings of this halide-intermediate interaction have been elegantly reported, but more studies are still needed to reach a definite conclusion. Strasser *et al.* proposed that the interaction between Cu and I^−^ favors the protonation of *CO (asterisk (*) denotes the adsorbed species) to produce CH_4_.^26^ On the other hand, Cuenya and colleagues revealed that the formation of *COOH is promoted by specific adsorption of halide ions, which improves the activity towards C_2+_ products.^13^ Moreover, Yeo *et al.* found that the specific adsorption of halide ions facilitates the formation of *CO, which then promotes the production of C_2+_ in the order of I^−^ > Br^−^ > Cl^−^ > ClO_4_^−^.^17^ Besides the role of specific adsorption, halide ions are also able to provide a high local pH, thereby promoting the formation of C_2+_.^16,20^ Even though the above studies provide comprehensive views of the effects of halide ions on the CO_2_RR, how the specific adsorption of halide ions influences the CO_2_RR process is still unclear. These different results probably arise from the entangled influences of the local pH, morphology of catalysts and/or concentration of the aqueous electrolyte with the introduction of halide ions into aqueous electrolytes, which have been well studied on the CO_2_RR.^11,15,16,24,25^ Therefore, in order to elucidate the role of specific adsorption of halide ions in the CO_2_RR, the local pH, the morphology and the cation concentration should be well controlled.

This paper describes a facile pre-reduction method in aqueous KX (X = Cl, Br, I) electrolytes to immobilize the rapidly evolving morphology of Cu-based electrocatalysts for the CO_2_RR. A non-buffered, non-specifically adsorbed and stable K_2_SO_4_ supporting electrolyte is used to further immobilize the obtained stable morphology, enable comparable change of local pH and maintain cation concentration during the CO_2_RR, which enables the exploration of the specific adsorption role of halide ions in the CO_2_RR. Furthermore, *in situ* attenuated total reflection surface enhanced infrared absorption spectroscopy (ATR-SEIRAS) shows that the adsorbed halide ions enhance *CO adsorption over Cu, thus promoting the kinetics of C–C coupling. At the same time, the faradaic efficiencies (FEs) toward C_2_H_4_ and C_2_H_5_OH can be improved with increasing halide anion concentration. The optimized C_2+_ FE of 84.5% is achieved in an aqueous 1.0 M KI electrolyte due to the strong specific adsorption of I^−^ over Cu.

## Experimental

### Catalyst synthesis and electrode preparation

CuO nanosheets (CuO-NSs) were synthesized *via* a simple hydrolysis method. In a typical synthesis, 35 mL 1 M NaOH was heated to 80 °C, after which, 2 mL 2.5 M Cu(NO_3_)_2_ was poured into heated NaOH solution immediately with stirring for 5 min until the solution turned into a black one. Then black particles were collected by using a centrifuge and washed with water and ethanol several times. The obtained particles were dried at 80 °C for 10 h. The catalyst inks were prepared by mixing CuO-NSs (16 mg), ethanol (1 mL) and Nafion solution (5 wt%, 40 μL) under sonication for 60 min. Next, the catalyst ink (30 μL) was added dropwise onto the surface of a glassy carbon electrode with a geometric area of 0.5 cm^2^.

### Characterization

X-ray diffraction (XRD) was performed by using a Bruker D8 with Cu Kα radiation (*λ* = 1.5418 Å) over a 2*θ* range of 30–80° at a scanning speed of 8° min^−1^. Transmission electron microscopy (TEM) images were recorded on a JEOL JEM-2100F using a Tecnai G2 F20 microscope with an acceleration voltage of 200 kV. Scanning electron microscopy (SEM) images were recorded on a Hitachi S-4800 with an acceleration voltage of 5 kV.

### *In situ* Raman spectroscopy

*In situ* Raman spectroscopy was carried out in a custom-designed H-type cell using a confocal Raman spectrometer (HORIBA, LabRAM HR Evolution). Copper foil coated with CuO-NSs was used as the working electrode with an exposed circular geometric surface area of ∼1 cm^2^. A platinum wire and an Ag/AgCl electrode (saturated KCl, Gaossunion Co., Ltd., Tianjin) were used as the counter and the reference electrode, respectively. 1.0 M KHCO_3_ aqueous solution was used as the anolyte and *x* M K_2_SO_4_ + *y* M KX (X = Cl, Br, I, 2*x* + *y* = 1) (Sigma Aldrich, 99%) was used as the catholyte and a bipolar membrane (FBM-PK) was used to separate the cathode and anode chambers. The excitation wavelength source was a visible light laser (532 nm). A water immersion objective lens (LUMFL, Olympus, 60×, numerical aperture: 1.10) was used to focus and collect the incident and scattered laser light. The Raman signal was recorded before, during and after applying potential, using a homemade electrochemical cell. The spectra were collected at OCP or under applied constant current density (−1 mA cm^−2^). Electrochemical measurements were carried out with a potentiostat (CompactStat.e20250, IVIUM).

### *In situ* ATR-SEIRAS experiments

*In situ* ATR-SEIRAS was performed with an attenuated total reflectance (ATR) configuration. A three-electrode configuration (Si prism, Ag/AgCl electrode and IrO_2_ mesh were used as the working electrode (WE), reference electrode (RE) and counter electrode (CE), respectively) was utilized in an H-type cell for electrochemical experiments. This cell is integrated into a FTIR (is50, Nicolet) spectrometer with a 60° incident angle (VeeMax, PIKE Technology). Spectra are presented in absorbance, with positive and negative peaks showing an increase and decrease in the signal, respectively. 1.0 M KHCO_3_ aqueous solution was used as the anolyte and *x* M K_2_SO_4_ + *y* M KX (X = Cl, Br, I, 2*x* + *y* = 1) (Sigma Aldrich, 99%) was used as the catholyte. A bipolar membrane (FBM-PK) was used to separate the cathode and anode chambers. The Si prism was covered with a gold film by chemical deposition and more details about the preparation process could be found in Deng.^27^ The enhanced signal for adsorbed intermediate species comes from the Au nanofilm in ATR-SEIRAS. To rule out the influence of the Au nanofilm, the same Au nanofilm covered Si wafer was used in *x* M K_2_SO_4_ + *y* M KI (*x* = 0.5, 0.33, 0.167, 0, 2*x* + *y* = 1). Two other Au nanofilm covered Si wafers were also used in *x* M K_2_SO_4_ + *y* M KBr (*x* = 0.5, 0.33, 0.167, 0, 2*x* + *y* = 1) and *x* M K_2_SO_4_ + *y* M KCl (*x* = 0.5, 0.33, 0.167, 0, 2*x* + *y* = 1), respectively. The same Au nanofilm covered by Si wafer was used when studying the same halide ion, and the Au nanofilm will not react with the halide ions under the CO_2_RR conditions. Thus the influence of the Au nanofilm was excluded. The catalyst suspension (16 mg mL^−1^) was added dropwise onto the Si surface covered with the Au nanofilm. Later, 20 mL aqueous electrolyte was bubbled with CO_2_ for 30 min before the test. The Si wafer loaded with catalysts was firstly pre-reduced in 0.2 M KX for 10 min to prevent the catalyst from falling off when −1.25 V is directly applied in a high concentration electrolyte, after which it was further reduced in 1.0 M KX for 20 min at −1.25 V *vs.* RHE. The background was taken at +0.1 V *vs.* RHE in an Ar saturated aqueous electrolyte. All spectra were collected at a resolution of 4 cm^−1^. Electrochemical measurements are carried out with a potentiostat (CompactStat.e20250, IVIUM).

### Electrochemical measurements

All electrochemical CO_2_ reduction experiments (CO_2_RR) were performed in a gas-tight H-type cell with two chambers separated using a three-electrode system connected to an electrochemical workstation (IVIUM4 Vertex). A glassy carbon electrode (0.5 cm^2^) covered with catalysts, 2 × 2 cm^2^ Pt foil and Ag/AgCl (with saturated KCl aqueous solution as the filling solution) were used as the WE, CE and RE, respectively. 1.0 M KHCO_3_ aqueous solution and *x* M K_2_SO_4_ + *y* M KX (X = Cl, Br, I, 2*x* + *y* = 1) (Sigma Aldrich, 99%) were used as the anolyte and catholyte, respectively. A bipolar membrane (FBM-PK) was used to separate the cathode and anode chambers. The cathode was connected to a mass flow controller (MC-Series, Alicat Scientific) and gas chromatograph (GC, Agilent 7890A) directly for on-line gas product detection. Before the start of the reaction, the aqueous electrolyte in the cathode was purged with CO_2_ (≥99.995%) for 30 min to achieve CO_2_ saturation and remove air in the system. During the measurement, the CO_2_ gas flow rate was controlled using a mass flow controller and set to 20 standard cubic centimeters per minute (sccm). Electrolysis experiments were conducted using chronoamperometry controlled with an electrochemical workstation. The cathode potentials were measured against an external reference electrode. For each potential, gas products were quantified over a period of 900 s. The gas products were analyzed by online gas chromatography (GC7890B, Agilent Technologies, Inc.) every 15 min. A thermal conductivity detector (TCD) connected to a MolSieve 5A packed column (Agilent Technologies, Inc.) was used to detect H_2_, O_2_, and N_2_ and a back flame ionization detector (FID) connected to a Porapak Q packed column (Agilent Technologies, Inc.) was used to detect CO. A front FID connected to an HP-PLOT Al_2_O_3_ capillary column (Agilent Technologies, Inc.) was used to detect hydrocarbons (C_1_–C_3_). Ar was used as the carrier gas. After passing through the reactor, the gas was allowed to flow directly into the gas sampling loop of the gas chromatography for online gaseous product analysis. Alcohols were analyzed with a GC (Shimadzu 2010 plus) equipped with a fused silica capillary column and FID when the amount of coulomb accumulated in the aqueous electrolyte reached 5 C mL^−1^.

## Results and discussion

### Morphology evolution of the catalyst in different aqueous electrolytes

**Fig. 1:**
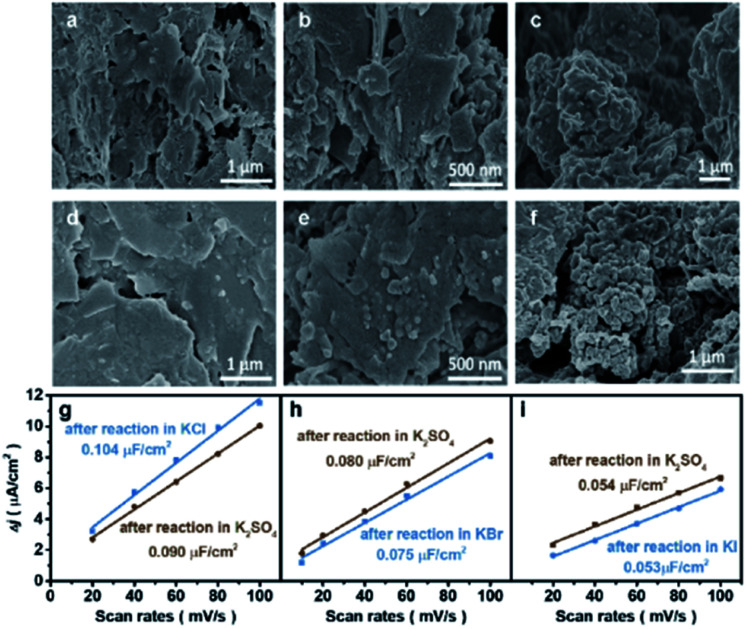
Morphology and ECSA of catalysts after reaction in various aqueous electrolytes. SEM images and ECSA of catalysts acquired after pre-reduction in 0.2 M KX at −1.25 V *vs.* RHE for 10 min, and then in various aqueous electrolytes at −1.25 V *vs.* RHE with comparable reaction durations: (a–c) 30 min reaction in 1.0 M KCl, KBr, and KI, respectively, (d–f) 30 min reaction in 1.0 M KCl, KBr, and KI, respectively, followed by another 30 min reaction in 0.5 M K_2_SO_4_, (g–i) blue: 30 min reaction in 1.0 M KCl, KBr, and KI, respectively, and brown: 30 min reaction in 1.0 M KCl, KBr, and KI, respectively, followed by another 30 min reaction in 0.5 M K_2_SO_4_.

The morphological changes of Cu induced by halide ions were investigated by the microscope technique. Oxide-derived Cu was adopted due to its high performance for the CO_2_RR to C_2+_, where CuO was synthesized using a simple hydrolysis method (details in the Experimental section). The as-synthesized CuO exhibited a nanosheet structure according to scanning electron microscopy (SEM), atomic force microscopy (AFM) and transmission electron microscopy (TEM) (Fig. S1†). However, the morphology of the obtained CuO-NSs was drastically different after being reduced in different aqueous KX electrolytes. The CuO-NS catalysts maintain the nanosheet structures after reduction in an aqueous KCl electrolyte (Fig. 1a) compared with the as-prepared catalyst (Fig. S1†), while the formation of nanoparticles is observed after reduction in an aqueous KBr electrolyte (Fig. 1b). The nanosheet structure is severely destructed accompanied by the formation of aggregated nanoparticles after reduction in KI (Fig. 1c). Similar results are further confirmed by the TEM images (Fig. S2†), where the degree of structural reconstruction follows the trend of KCl < KBr < KI. These results demonstrate that the structure of Cu-based catalysts may change drastically during the CO_2_RR in different aqueous KX electrolytes.^23^ Therefore, it is not possible to evaluate the CO_2_RR performance directly in different kinds of aqueous KX electrolytes, and the influence of specific adsorption of halide ions on the CO_2_RR should be evaluated in the same kind of aqueous KX electrolyte with varying halide ion concentrations. Thus, an aqueous supporting electrolyte is needed to maintain a constant cation (specifically K^+^) concentration,^15^ with halide ion concentrations as the only variable. An aqueous KHCO_3_ electrolyte, the most widely used aqueous HCO_3_^−^ buffer electrolyte, could be used as the supporting electrolyte to compare with other studies, where the concentration of KHCO_3_ could be controlled according to halide ions to maintain a constant cation concentration. However, the morphologies of Cu-based catalysts pre-reduced in an aqueous KI electrolyte experience a drastic change after reaction in an aqueous electrolyte containing KHCO_3_ (Fig. S3†), leading to the change in CO_2_RR activity and selectivity (Fig. S4†).^23^ Therefore, the reconstruction of catalysts in aqueous KX and KHCO_3_ mixed electrolytes during the CO_2_RR makes it difficult to elucidate the influence of specific adsorption of halide ions towards the CO_2_RR.

In order to elucidate the effects of the specific adsorption of halide ions on the CO_2_RR, K_2_SO_4_ with good solubility was adopted as the supporting electrolyte (replacing KHCO_3_) to immobilize the morphology of Cu after reconstruction in KX. As a non-buffered electrolyte, the SO_4_^2−^ anions in K_2_SO_4_ are stable under cathodic conditions, with chemical properties similar to those of halide ions except for specific adsorption on the catalyst. Compared with KHCO_3_, the morphologies obtained after pre-reduction in aqueous KX electrolytes could be immobilized after reaction in non-specifically adsorbed K_2_SO_4_, as demonstrated by the SEM (Fig. 1d–f) images and the roughness (Fig. 1g–i). The electrochemically active surface area (ECSA) was also obtained during reaction at different duration intervals in 0.5 M K_2_SO_4_ solution (Fig. S5†). It can be found that the ECSA of the electrode are the same during reaction from 10 min to 70 min (Fig. S5†). Thus, aqueous KX and K_2_SO_4_ mixed electrolytes with a constant cation concentration can be used to immobilize these morphologies during the CO_2_RR (Fig. S6†). In addition to SEM, TEM was also used to characterize the structure of catalysts. The nanosheet structure could be maintained after pre-reduction and reaction in the KCl electrolyte. Moreover, the nanosheet structure of the catalyst obtained after pre-reduction and reaction in the KCl electrolyte is still retained after reaction in *x* M K_2_SO_4_ + *y* M KCl (2*x* + *y* = 1, *x* = 0.167, 0.33, 0.5) (Fig. S7†). However, small particles were formed on nanosheets when CuO–NSs were pre-reduced and reacted in the KBr electrolyte. In the subsequent reaction in *x* M K_2_SO_4_ + *y* M KBr (2*x* + *y* = 1, *x* = 0.167, 0.33, 0.5) electrolytes, there are still particles coated on the nanosheets (Fig. S8†). Due to the fact that the specific adsorption of I^−^ is the strongest among these halide ions, the nanosheets were totally deconstructed and aggregated particles were formed after pre-reduction and reaction in the KI electrolyte. These particles were also maintained after reacting in *x* M K_2_SO_4_ + *y* M KI (2*x* + *y* = 1, *x* = 0.167, 0.33, 0.5) (Fig. S9†). As a result, the influence of morphologies is well eliminated when the CuO-NS catalysts are pre-reduced in aqueous KX with K_2_SO_4_ used as the supporting electrolyte.

### CO_2_RR performance in aqueous KX and K_2_SO_4_ mixed electrolytes

The same protocol would be used in the following studies to immobilize the surface structures of Cu reconstructed in aqueous KX electrolytes. To be specific, the catalysts were consecutively pre-reduced in CO_2_-saturated aqueous 0.2 M KX and 1.0 M KX electrolytes for 10 min and 20 min, respectively, at −1.25 V *vs.* RHE. After that, a stable morphology was obtained and aqueous K_2_SO_4_ was used as the supporting electrolyte to immobilize the obtained morphologies. Compared with the commonly used KHCO_3_ buffer, aqueous KX is a non-buffering electrolyte will result in a drastic change of local pH during the CO_2_RR. Thus, another non-buffering electrolyte, K_2_SO_4_, was used as the supporting electrolyte to enable a similar change of the local pH comparable with KX during the CO_2_RR. Thus, the comparable change of local pH in *x* M K_2_SO_4_ + *y* M KX (2*x* + *y* = 1, *x* = 0, 0.17, 0.33, 0.5) with different *x* values would guarantee the correct interpretation of specific adsorption of halide ions with varying local pH (Fig. S10†).^28^ However, if the buffered KHCO_3_ was used as the supporting electrolyte, the change of local pH values would be limited, leading to different and incomparable local pH values with KX at different concentrations.^19^ Thus, using aqueous K_2_SO_4_ (compared with KHCO_3_) as the supporting electrolyte could immobilize the morphology of catalysts and enable comparable change of local pH.

Although it is possible to immobilize the catalyst morphology and enable comparable change of local pH, the concentration of cations has to be considered when investigating the influence of changes in halide ion concentration on the CO_2_RR.^15^ In general, if KX solution is used alone, the concentration of cations will change with changing concentration of halide ions, and the change of cation concentration always leads to the variation of current density.^15^ With the increase of cation concentration, the partial current density of the C_2+_ product increases, while its selectivity remains unchanged (Fig. S11†). Thus, in order to maintain the same cation concentration (1 M), the CO_2_RR performance was evaluated over the pre-reduced electrode by adjusting the ratio of K_2_SO_4_ to KX aqueous electrolyte, where the composition of the aqueous electrolyte was *x* M K_2_SO_4_ + *y* M KX (2*x* + *y* = 1, *x* = 0, 0.17, 0.33, 0.5).

**Fig. 2:**
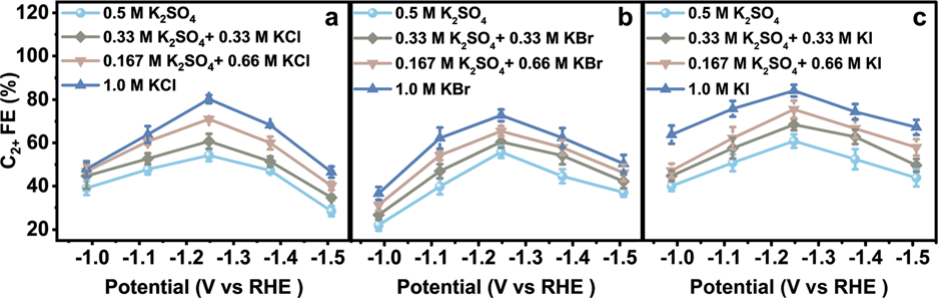
Catalytic performances in various aqueous electrolytes. C_2+_ FE in aqueous (a) K_2_SO_4_ and KCl mixed electrolytes, (b) K_2_SO_4_ and KBr mixed electrolytes, and (c) K_2_SO_4_ and KI mixed electrolytes at various applied potentials. The catalysts were pre-reduced in aqueous 0.2 M KX for 10 min and 1.0 M KX for 20 min, respectively, at −1.25 V *vs.* RHE.

The electrodes were rinsed with deionized water thoroughly when they were transferred between different aqueous electrolytes for CO_2_RR evaluation. The obtained CO_2_RR activity and selectivity demonstrate the significant influence of halide ions on the CO_2_RR. The C_2+_ FE in *x* M K_2_SO_4_ + *y* M KX indicates that C_2+_ FE increases with increasing concentration of halide ions (Cl^−^, Br^−^, and I^−^) (Fig. 2). From −0.99 to −1.51 V *vs.* RHE, the C_2+_ FE reaches the maximum value around −1.25 V *vs.* RHE in all aqueous electrolytes. Other products in aqueous KX and K_2_SO_4_ mixed electrolytes are also compared (Fig. S12–S17†). An optimized C_2+_ FE of 84.5% could be achieved in 1.0 M KI at −1.25 V *vs.* RHE with a C_2+_ partial current density of 36.3 mA cm^−2^. A long-term stability test was also conducted in 1.0 M KI at −1.25 V *vs.* RHE (Fig. S18†) with ethylene diamine tetraacetic acid (EDTA) added in an aqueous electrolyte as a reliable impurity scavenger.^29^ The C_2_H_4_ FE remains above 50% during an 8 hour stability test. In summary, the production of C_2+_ could be enhanced with the increasing concentration of Cl^−^, Br^−^ and I^−^, which may be attributed to the increased CuX species or specific adsorption of halide ions on the catalyst when the halide ion concentration was increased.

### Investigation of CuX species in the catalyst

**Fig. 3:**
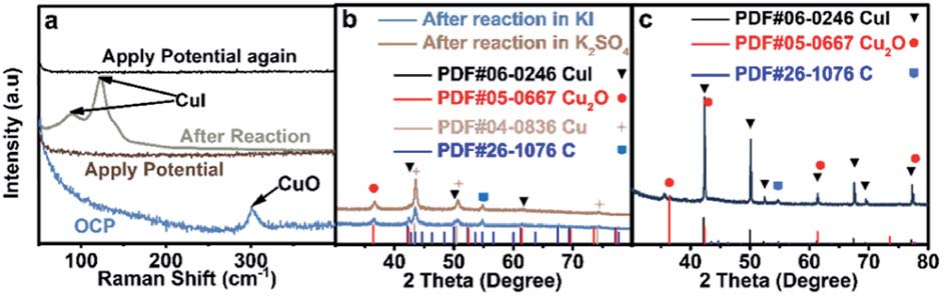
Analysis of catalyst species. (a) *In situ* Raman before, during and after applying potential; XRD patterns of catalysts acquired after reduction at −1.25 V *vs.* RHE in various aqueous electrolytes with comparable reaction durations: (b) blue: 30 min reaction in 1.0 M KI, and brown: 30 min reaction in 1.0 M KI, followed by another 30 min reaction in 0.5 M K_2_SO_4_, and (c) 30 min reaction in 0.5 M K_2_SO_4_, followed by immersed in 1.0 M KI without applying bias.

The interaction between I and Cu is the strongest among the above stated three halide ions, which is verified by the most prominent morphological change of CuO in an aqueous KI electrolyte (Fig. 1c). Moreover, the electron transfer between I and Cu is also significant since the formation of CuI could significantly stabilize the reaction intermediates that enhance the C_2+_ production pathway.^25^ Although CuI is observed by Raman (Fig. 3a)^30^ and XRD (Fig. 3b) after reaction, which may be formed during the oxidation of metallic Cu in the presence of KI (Fig. 3c), it cannot be concluded that CuI species indeed exist during the reaction. The *in situ* Raman spectrum was recorded when the applied current density is −1 mA cm^−2^ to eliminate the influence of bubbles produced on the cathode during reaction, and the applied potential was around −0.38 V *vs.* RHE (Fig. S19†). The *in situ* Raman spectrum confirmed that CuI species disappeared upon applying bias, according to the absence of two peaks at 89 cm^−1^ and 124 cm^−1^ belonging to CuI (Fig. 3a). The silent Raman spectrum in the range of 50 cm^−1^ to 400 cm^−1^ also indicated that CuO species were reduced under reaction conditions (Fig. 3a). In addition to CuI, the main species of the catalysts are all metallic Cu after reaction in *x* M K_2_SO_4_ + *y* M KI (2*x* + *y* = 1, *x* = 0, 0.17, 0.33, 0.5) electrolytes (Fig. S20†). Moreover, Cu(111)/Cu(100) are similar after reaction in an electrolyte containing the same halide ion (Table S3†). Aside from Cu and CuI, the XRD patterns of Cu_2_O (Fig. 3b and S20†) could be attributed to the oxidation of metallic Cu catalysts upon sample transfer under ambient conditions, which is confirmed by the absence of Cu_2_O signals according to *in situ* Raman under reaction conditions (Fig. 3a). Therefore, it could be concluded that the main difference between electrodes in different aqueous electrolytes with varying concentration of KX is the amount of specific adsorption of halide ions on catalysts, which may influence the selectivity of C_2+_ products.^26^

### Mechanistic study by *in situ* ATR-SEIRAS

**Fig. 4:**
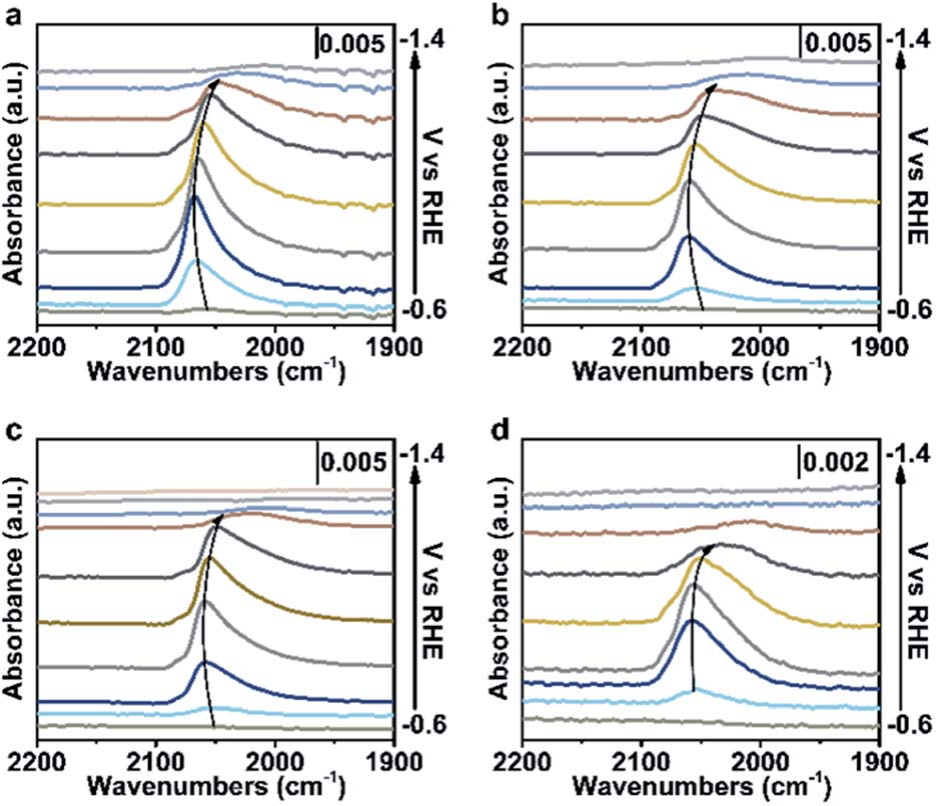
Spectroscopic investigations in different electrolytes. *In situ* ATR-SEIRAS spectra recorded at various potentials in CO_2_-saturated aqueous (a) 0.5 M K_2_SO_4_, (b) 0.33 M K_2_SO_4_ + 0.33 M KI, (c) 0.167 M K_2_SO_4_ + 0.66 M KI and (d) 1.0 M KI electrolytes with catalysts loading on a Si wafer covered by Au. The catalysts were pre-reduced in aqueous 0.2 M KI for 10 min, and 1.0 M KI for 20 min, respectively, at −1.25 V *vs.* RHE.

To further verify our proposed mechanism for halide ions over the selectivity of C_2+_ products, *in situ* ATR-SEIRAS was adopted to study the effect of halide ions on intermediate species in CO_2_ saturated aqueous *x* M K_2_SO_4_ + *y* M KX (2*x* + *y* = 1, *x* = 0, 0.17, 0.33, 0.5) electrolytes (Fig. 4, S21 and S22†). The catalyst was firstly reduced in 0.2 M KX for 10 min and then in 1.0 M KX for 20 min just like in the activity test to obtain a stable morphology and eliminate the influence of other factors induced by halide ions during the reconstruction process. Stretching bands between 2000 cm^−1^ and 2100 cm^−1^ in all aqueous electrolytes are observed (Fig. 4, S21 and S22†), corresponding to the stretching band of atop-bound CO (*CO) on the Cu surface.^31^

**Fig. 5:**
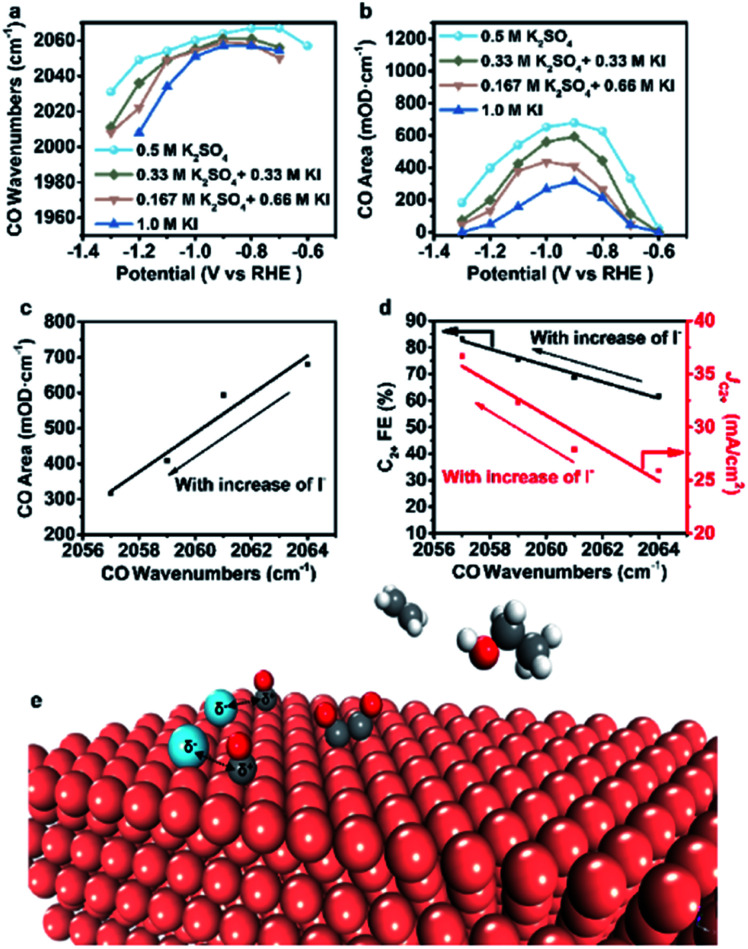
Analysis of spectral results and the proposed reaction mechanism. (a) CO wavenumbers and (b) CO areas vary with potential in different aqueous electrolytes. (c) Variation of CO areas with CO wavenumbers at −0.9 V *vs.* RHE as the I^−^ concentration varies. (d) Variation of C_2+_ FE and C_2+_ partial current density at −1.25 V with CO wavenumbers at −0.9 V as the I^−^ concentration varies. (e) A proposed reaction mechanism for the enhancement of the CO_2_RR to C_2+_ induced by halide ion adsorption. Blue, halide ions; grey, carbon; red, oxygen; brick-red, copper; white, hydrogen.

However, the accurate wavenumbers slightly change in different aqueous electrolytes (Fig. 5a). Compared with the wavenumbers of *CO in K_2_SO_4_, the *CO band exhibits a distinct red-shift at all potentials in aqueous electrolytes containing halide ions. The wavenumbers of *CO decrease with increasing halide ion concentration due to the enhanced specific adsorption of halide ions (Fig. 5a, S23a and S24a†),^32^ which indicates the stronger adsorption of *CO in KX compared to that in K_2_SO_4_.^6,33^ In addition, the intensity (milli-optical density (mOD)) and peak area of the *CO band, which are proportional to the surface coverage of *CO, decrease as the concentration of halide ions increases under the same potential (Fig. 5b, S23b and S24b†). From aqueous 0.5 M K_2_SO_4_ to 1.0 M KI electrolytes, the wavenumbers of *CO decrease from 2064 cm^−1^ to 2056 cm^−1^ while the peak areas decrease from 679.3 mOD cm^−1^ to 315.2 mOD cm^−1^ at −0.9 V *vs.* RHE (Fig. 5c). In the meanwhile, the C_2+_ FE increases from 61.8% to 84.5% (Fig. 5d). Similar trends are also observed in aqueous KCl/KBr and K_2_SO_4_ mixed electrolytes (Fig. S23c, d and S24c, d†). Due to the specific adsorption of halide ions, negative charges from X^−^ may transfer to *CO,^25^ which may lead to an increase in the extent of *CO dπ–2π* back-donation, resulting in the stronger adsorption of *CO as demonstrated by the lower wavenumbers of *CO. Meanwhile, the peak area of *CO decreases with increasing concentration of halide ions, indicating faster *CO depletion, suggesting enhanced C–C coupling. Thus, it could be concluded that the specific adsorption of halide ions promotes the adsorption of *CO in aqueous X^−^ containing electrolytes, and more electrons are transferred to *CO with the increase of X^−^, leading to faster C–C coupling kinetics (Fig. 5e).

## Conclusions

In summary, this paper elucidates the key role of specific adsorption of halide ions in the CO_2_RR by pre-reducing catalysts in aqueous KX electrolytes and using aqueous K_2_SO_4_ as the supporting electrolyte. The effect of specific adsorption of halide ions and other factors (morphology, local pH and cation concentration) induced by the introduction of halide ions in aqueous electrolytes are successfully decoupled by this method. Stable morphologies were obtained by pre-reducing catalysts in aqueous KX electrolytes. Although different halide ions induced the generation of different morphologies after pre-reduction, an aqueous K_2_SO_4_ supporting electrolyte was used to immobilize these reconstructed morphologies. At the same time, using aqueous K_2_SO_4_ as the supporting electrolyte could also enable comparable change of local pH and maintain cation concentration. The selectivity and activity toward C_2_H_4_ and C_2_H_5_OH are enhanced with increasing halide ion concentrations without the interference of other factors. The C_2+_ FE and partial current density are linearly dependent on the concentration of halide ions, mainly due to the stronger adsorption of *CO induced by the specific adsorption of halide ions, as evidenced by *in situ* ATR-SEIRAS. The enhancement of *CO adsorption further promotes the C–C coupling kinetics, leading to the accelerated production of C_2_H_4_ and C_2_H_5_OH.

## Data availability

The article and ESI† contain all the experimental and computational data.

## Author contributions

J. L. G. supervised the project. J. L. G., T. W., and T. H. Y. conceptualized the project. T. H. Y. synthesized CuO-NSs and conducted the CO_2_RR performance tests. T. H. Y., G. Z. and W. Y. D. designed the experiment and analyzed the related data. D. F. C. and H. G. constructed the reaction model. J. Z. and J. Y. conducted related characterization. All the authors participated in the writing of the manuscript.

## Conflicts of interest

There are no conflicts to declare.

## Supplementary Material

SC-013-D2SC02689A-s001
