# Supplementary material for: The effect of specific adsorption of halide ions on electrochemical CO2 reduction
Source: Chem Sci. 2022 Jun 28;13(27):8117–23. doi: 10.1039/d2sc02689a (PMC9278460; doi:10.1039/d2sc02689a)
Supplement: SC-013-D2SC02689A-s001 [file SC-013-D2SC02689A-s001.pdf]

Electronic Supplementary Material (ESI) for Chemical Science.
This journal is © The Royal Society of Chemistry 2022

Supplementary Information

The Effect of Specific Adsorption of Halide Ions towards Electrochemical CO_2 Reduction

Tenghui Yuan,[abc] Tuo Wang,[abc] Gong Zhang,[abc] Wanyu Deng,[abc] Dongfang Cheng,[abc] Hui Gao,[abc] Jing Zhao,[abc] Jia Yu,[abc] Peng Zhang,[abc] Jinlong Gong*[abcd]

[a]School of Chemical Engineering and Technology, Key Laboratory for Green Chemical Technology of Ministry of Education, Tianjin University, Tianjin 300072, China.

[b]Collaborative Innovation Center for Chemical Science & Engineering (Tianjin), Tianjin 300072, China.

[c]Haihe Laboratory of Sustainable Chemical Transformations, Tianjin 300192, China.

[d]Joint School of National University of Singapore and Tianjin University, International Campus of Tianjin University, Binhai New City, Fuzhou 350207, China.

Corresponding Author

*E-mail: jlgong@tju.edu.cn

Materials

$Cu(NO_3)_2$ (99.5%), NaOH (99%), KI (99%), KCl (99%) and KBr (99%) were purchased from Tianjin Guangfu Technology Development Co. Ltd., $HAuCl_4{\cdot}3H_2O$ (99%), K_2SO_4 (99%) and NaOH (99%) were purchased from Aladdin Industrial Co. Ltd. H_2SO_4 (95-98%) was purchased from Sinopharm Chemical Reagent Co. Ltd. $Na_2S_2O_3$ (99.5%), and HF (40.0%) were purchased from J&K Scientific Ltd. NH_4F (96.0%), NH_4Cl (99.5%), $C_{10}H_{16}N_2O_8$ (EDTA, 99%) and ethanol (99.8%) were purchased from Tianjin Yuanli Technology Development Co. Ltd. (China). All chemical reagents were used as received without further purification. Al_2O_3 polishing powder (0.05 μm), 5 wt% Nafion solution and 99.999% purity carbon dioxide gas were purchased from Gaoss Union Technology Co. Ltd., Dupont and Air Liquide, respectively. All aqueous solutions used ultrapure (UP) water (18.25 MΩ·cm) as the solvent.

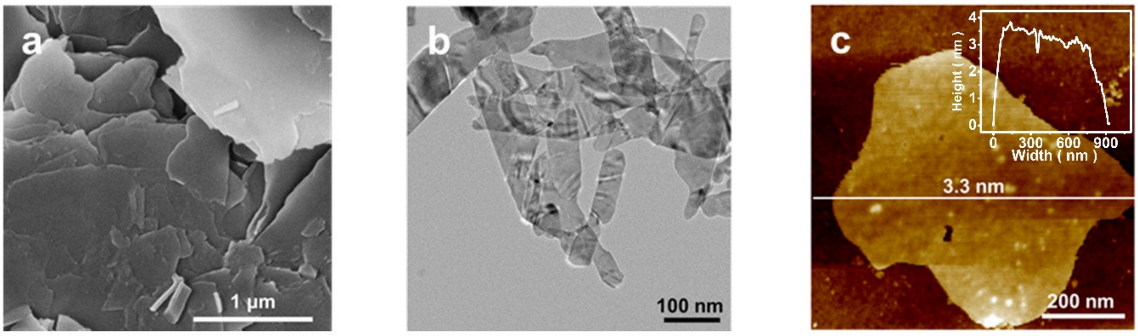

Figure S1. (a) SEM, (b) TEM and (c) AFM images of CuO.

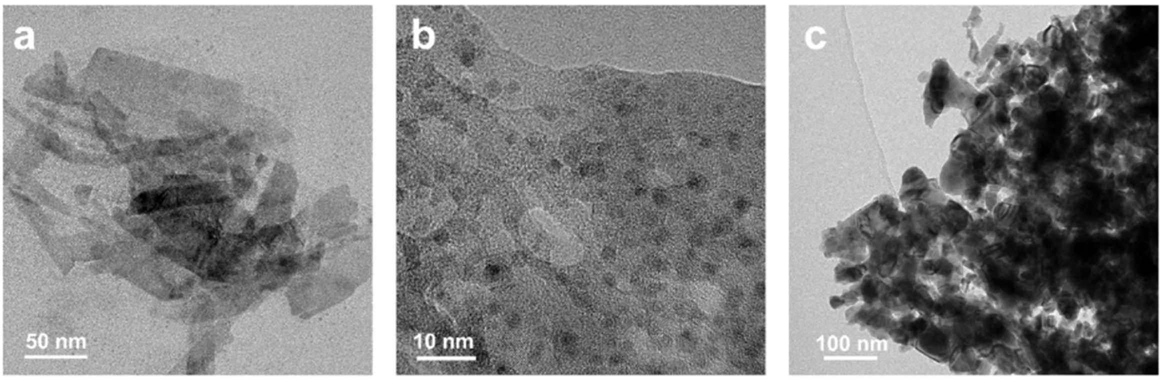

Figure S2. TEM images of catalysts acquired after pre-reduction in 0.2 M KX at −1.25 V vs RHE for 10 min, then in various aqueous electrolytes with comparable reaction durations: (a-c) 30 min reaction in 1.0 M KI, KBr, and KCl, respectively.

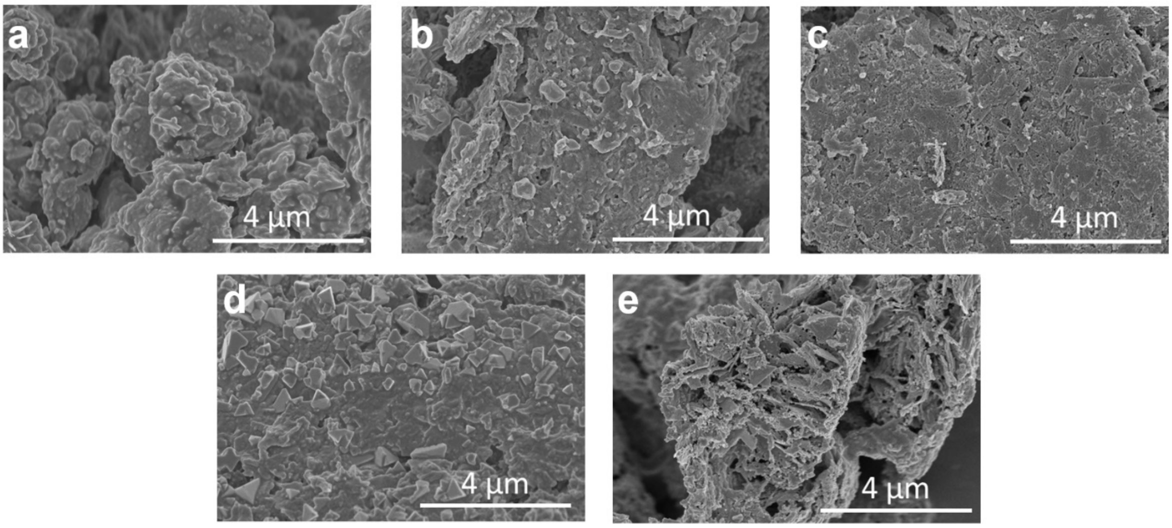

Figure S3. SEM images of catalysts acquired after reaction at −1.25 V vs RHE in various aqueous electrolytes with comparable reaction durations: (a) 30 min reaction in 1.0 M KI, (b) 30 min reaction in 1.0 M KI, followed by another 30 min reaction in 0.5 M KI + 0.5 M $KHCO_3$, (c) 30 min reaction in 1.0 M KI, followed by another 30 min reaction in 1.0 M $KHCO_3$, (d) 30 min reaction in 0.5 M KI + 0.5 M $KHCO_3$, (e) 30 min reaction in 1.0 M $KHCO_3$.

In order to investigate the influence of different morphologies induced by pre-reduction conditions, CO_2RR tests were conducted in aqueous 1.0 M $KHCO_3$ and 0.5 M KI + 0.5 M $KHCO_3$ mixed electrolytes with different pre-reduction conditions. The main product was H_2 (around 73%) and the C_2H_4 FE was only about 3% in 1.0 M $KHCO_3$ without pre-reduction (Figure S4a), while the H_2 decreased to around 57% and the C_2H_4 increased to over 10% in 1.0 M $KHCO_3$ with pre-reduction in 1.0 M KI (Figure S4b). Besides, pre-reduction also took an effect on product distribution when conducting CO_2RR in 0.5 M KI + 0.5 M $KHCO_3$ mixed electrolytes. The main product was H_2 and C_2H_4 when catalysts were pre-reduced in 1.0 M KI, and the selectivity of CH_4 was pretty low (Figure S4c). However, H_2 decreased and CH_4 increased apparently when CO_2RR was tested in 0.5 M KI + 0.5 M $KHCO_3$ mixed electrolytes directly without pre-reduction (Figure S4d)[1]. Therefore, the pre-reduction conditions play a key role in the product distribution due to different morphologies of catalysts induced by different pre-reduction conditions (Figure S3). Taking into consideration that halide ions induce drastic changes in morphology of catalysts, it is important to eliminate the influence of morphology when investigating specific adsorption of halide ions.

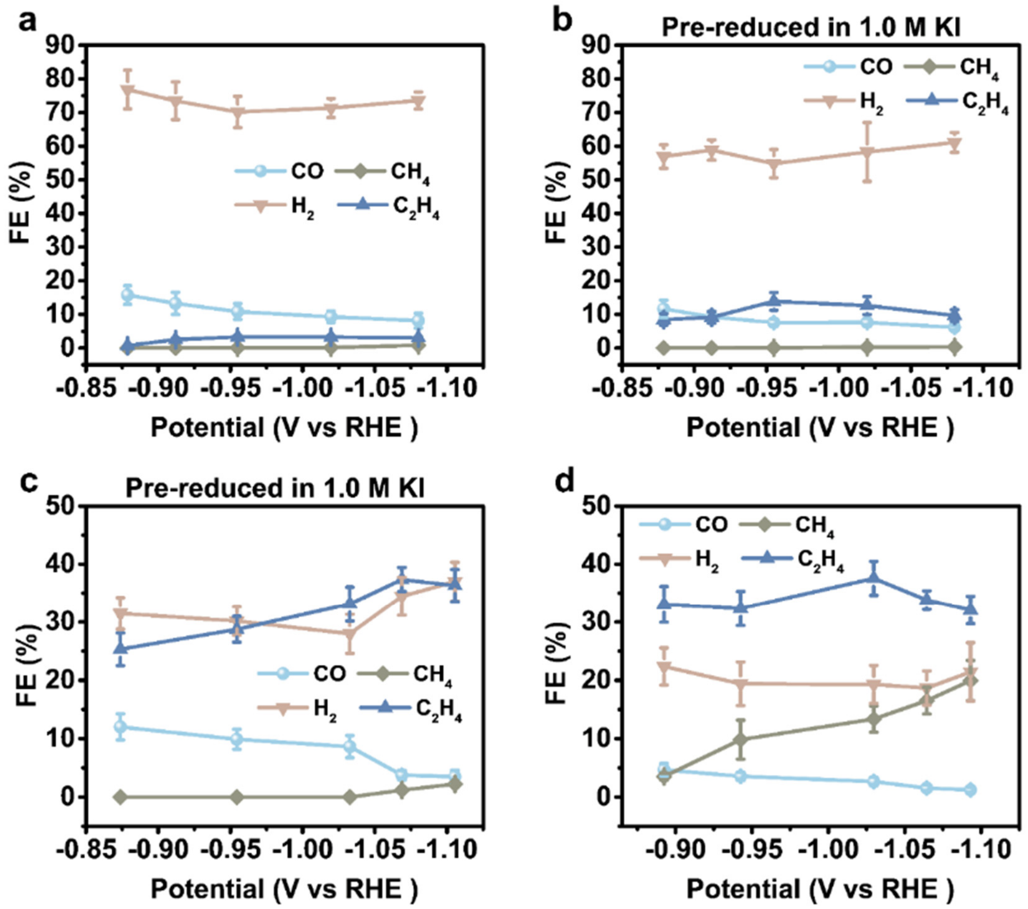

Figure S4. FE for CO, H_2, CH_4 and C_2H_4 in aqueous (a) 1.0 M $KHCO_3$ electrolyte without pre-reduction (b) 1.0 M $KHCO_3$ electrolyte with pre-reduction in 1.0 M KI, (c) 0.5 M $KHCO_3$ + 0.5 M KI mixed electrolyte with pre-reduction in 1.0 M KI and (d) 0.5 M $KHCO_3$ + 0.5 M KI mixed electrolyte without pre-reduction.

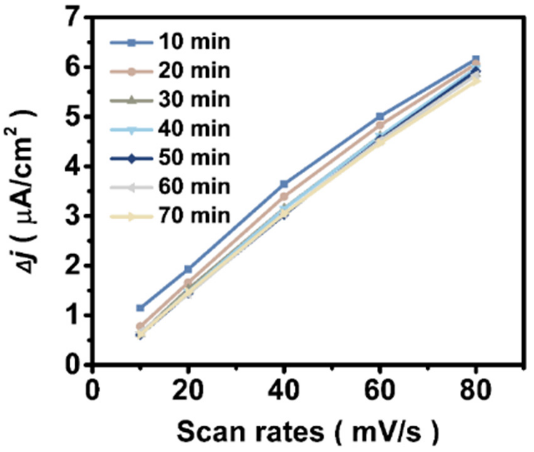

Figure S5. ECSA of catalysts acquired after CO_2RR reaction from 10 min to 70 min in 0.5 M K_2SO_4 at −1.25 V vs RHE.

The CuO nanosheets were firstly pre-reduced in 0.2 M KI at −1.25V vs RHE for 10 min to prevent the catalyst from falling off, after which, CO_2RR was conducted in CO_2 saturated 0.5 M K_2SO_4 for 70 min. The ECSA was tested every 10 min during CO_2RR reaction. In the process of ECSA test, the electrode was not taken out from the electrolyte, but electrolyte was saturated with Ar. Because the ECSA test process does not change the electrolyte, the ECSA test process does not affect the ECSA of catalysts. The procedure was also taken for ECSA test in other electrolytes.

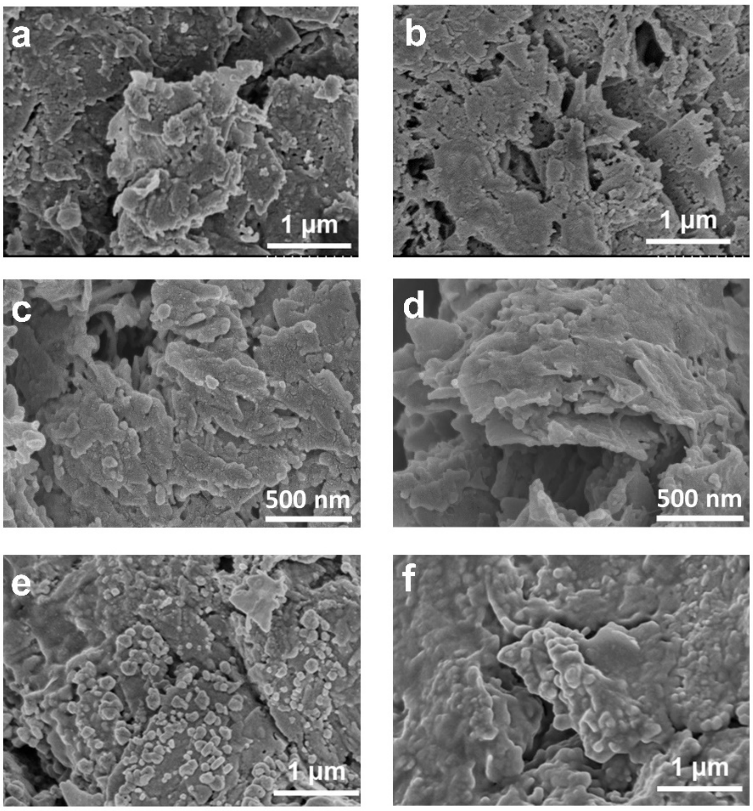

Figure S6. SEM images of catalysts acquired after pre-reduction in 0.2 M KX at −1.25 V vs RHE for 10 min, then in various aqueous electrolytes at −1.25 V vs RHE with comparable reaction durations: (a-b) 30 min reaction in 1.0 M KCl, followed by another 30 min reaction in 0.66 M KCl + 0.167 M K_2SO_4 and 0.33 M KCl + 0.33 M K_2SO_4, respectively, (c-d) 30 min reaction in 1.0 M KBr, followed by another 30 min reaction in 0.66 M KBr + 0.167 M K_2SO_4 and 0.33 M KBr + 0.33 M K_2SO_4, respectively, (e-f) 30 min reaction in 1.0 M KI, followed by another 30 min reaction in 0.66 M KI + 0.167 M K_2SO_4 and 0.33 M KI + 0.33 M K_2SO_4, respectively.

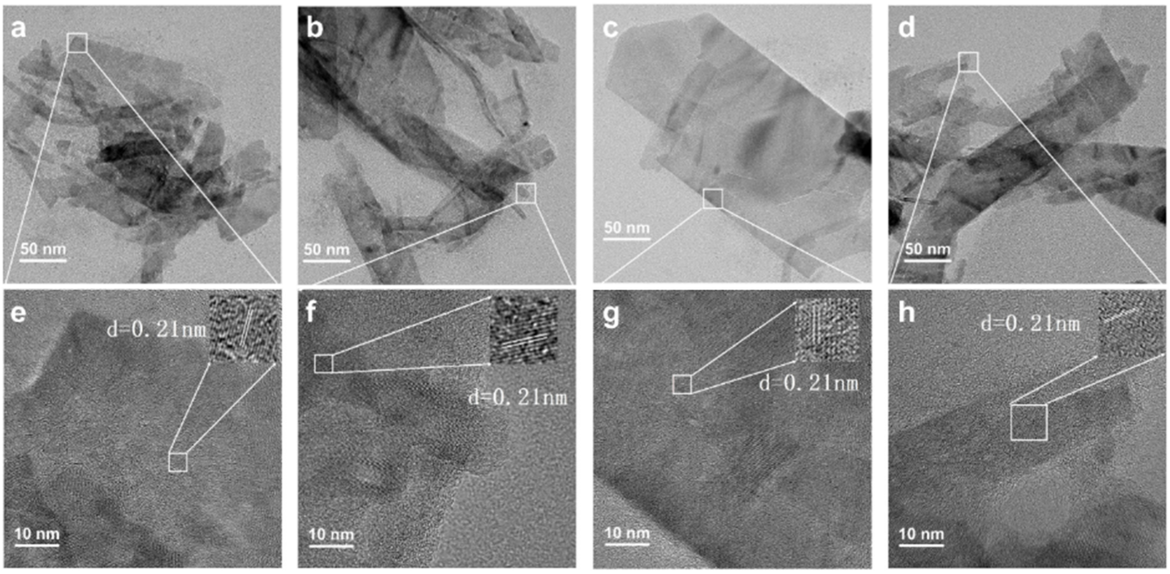

Figure S7. TEM images of catalysts acquired after pre-reduction in 0.2 M KCl at −1.25 V vs RHE for 10 min, and in various aqueous electrolytes at −1.25 V vs RHE with comparable reaction durations: (a) 30 min reaction in 1.0 M KCl, (b) 30 min reaction in 1.0 M KCl, followed by another 30 min reaction in 0.66 M KCl + 0.167 M K_2SO_4, (c) 30 min reaction in 1.0 M KCl, followed by another 30 min reaction in 0.33 M KCl + 0.33 M K_2SO_4, (d) 30 min reaction in 1.0 M KBr, followed by another 30 min reaction in 0.5 M K_2SO_4, (e-h) high resolution TEM of (a-d), respectively.

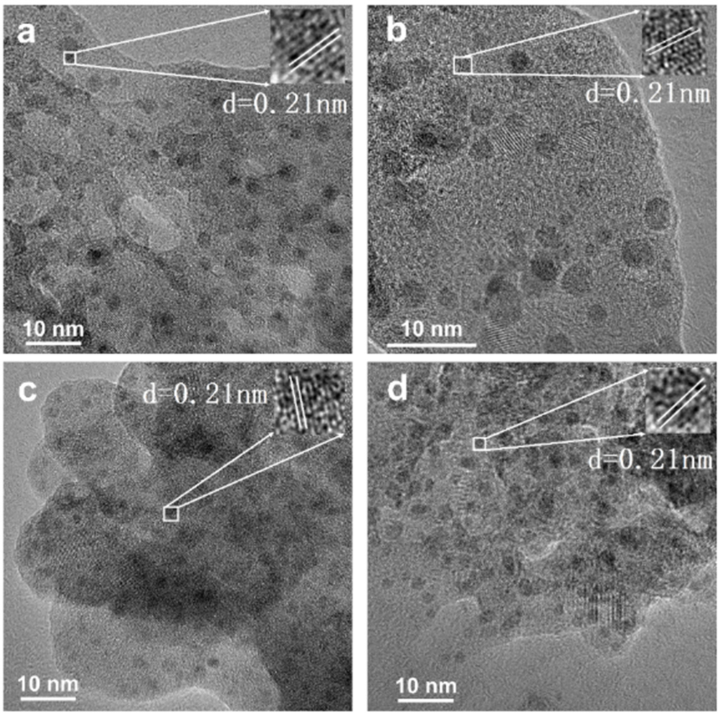

Figure S8. TEM images of catalysts acquired after pre-reduction in 0.2 M KBr at −1.25 V vs RHE for 10 min, and in various aqueous electrolytes at −1.25 V vs RHE with comparable reaction durations: (a) 30 min reaction in 1.0 M KBr, (b) 30 min reaction in 1.0 M KBr, followed by another 30 min reaction in 0.66 M KBr + 0.167 M K_2SO_4, (c) 30 min reaction in 1.0 M KBr, followed by another 30 min reaction in 0.33 M KBr + 0.33 M K_2SO_4, (d) 30 min reaction in 1.0 M KBr, followed by another 30 min reaction in 0.5 M K_2SO_4.

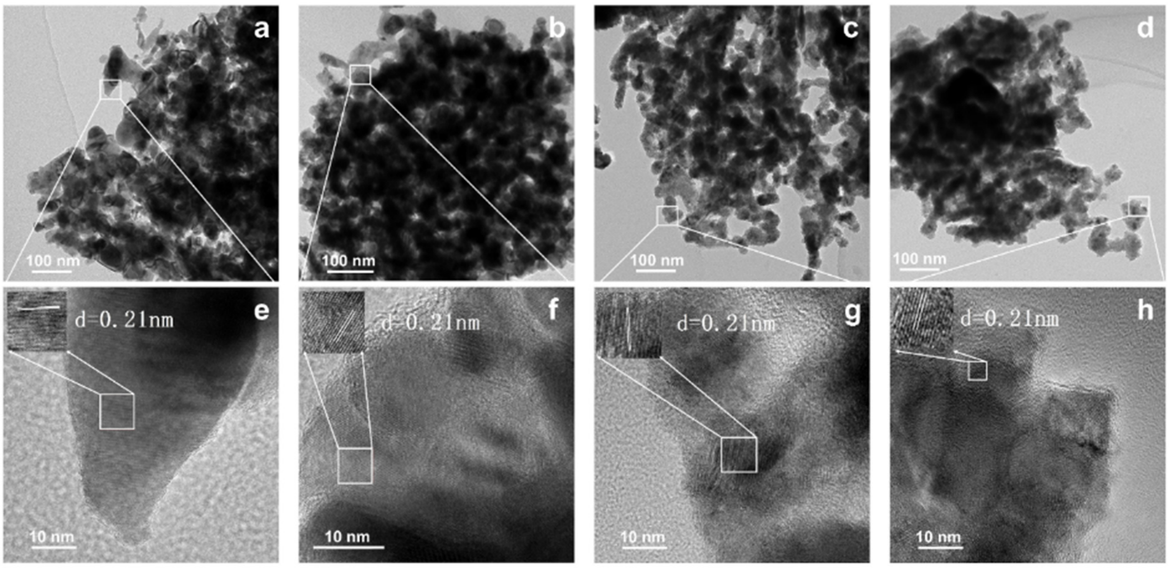

Figure S9. TEM images of catalysts acquired after pre-reduction in 0.2 M KI at −1.25 V vs RHE for 10 min, and in various aqueous electrolytes at −1.25 V vs RHE with comparable reaction durations: (a) 30 min reaction in 1.0 M KI, (b) 30 min reaction in 1.0 M KI, followed by another 30 min reaction in 0.66 M KI + 0.167 M K_2SO_4, (c) 30 min reaction in 1.0 M KI, followed by another 30 min reaction in 0.33 M KI + 0.33 M K_2SO_4, (d) 30 min reaction in 1.0 M KI, followed by another 30 min reaction in 0.5 M K_2SO_4, (e-h) high resolution TEM of (a-d), respectively.

Both aqueous K_2SO_4 and KX are non-buffered electrolytes, thus, the local pH would change drastically during CO_2RR in *x* M K_2SO_4 + *y* M KX ($2x + y = 1$, $x = 0, 0.17, 0.33, 0.5$). Besides, the similar current densities in these electrolytes confirmed the comparable change of local pH under the same potentials during CO_2RR (Fig. S10), which would guarantee the correct interpretation of specific adsorption of halide ions by excluding the interference of the local pH[2].

In CO_2 saturated electrolyte,

$$CO_2(aq) + H_2O \rightleftharpoons HCO_3^- + H^+ \quad (1)$$

The H^+ is consumed and OH^- is generated on the cathode. CO_2 and HCO_3^- may be neutralized by OH^- ion generated on the cathode surface by:

$$CO_2(aq) + OH^- \rightleftharpoons HCO_3^- \quad (2)$$

$$HCO_3^- + OH^- \rightleftharpoons CO_3^{2-} + H_2O \quad (3)$$

The forward and reverse reaction rate constants for equation 2 and 3 have been reported[2] and are summarized in Table S1:

Table S1. Reaction Rate Constants

Reaction	Forward reaction rate constant/M^{-1} s^{-1}	Reverse reaction rate constant/s^{-1}
2	$k_{1f} = 5.93 \times 10^3$	$k_{1r} = 1.34 \times 10^{-4}$
3	$k_{1f} = 1 \times 10^8$	$k_{1f} = 2.15 \times 10^4$

According to the electrode–electrolyte boundary layer in the CO_2 electroreduction system. Film theory is assumed to be applicable in the concentration boundary layer, and the velocity gradients or convective effects are assumed to be negligible. The equation can be established as follows:

$$\frac{\partial[CO_2(aq)]}{\partial t} = D_{CO_2}\frac{\partial^2[CO_2(aq)]}{\partial x^2} - [CO_2(aq)][OH^-]k_{1f} + [HCO_3^-]k_{1r}$$

$$\frac{\partial[HCO_3^-]}{\partial t} = D_{HCO_3^-}\frac{\partial^2[HCO_3^-]}{\partial x^2} + [CO_2(aq)][OH^-]k_{1f} - [HCO_3^-]k_{1r}$$

$$- [HCO_3^-][OH^-]k_{2f} + [CO_3^{2-}]k_{2r}$$

$$\frac{\partial[CO_3^{2-}]}{\partial t} = D_{CO_3^{2-}}\frac{\partial^2[CO_3^{2-}]}{\partial x^2} + [HCO_3^-][OH^-]k_{2f} - [CO_3^{2-}]k_{2r}$$

$$\frac{\partial[OH^-]}{\partial t} = D_{OH^-}\frac{\partial^2[OH^-]}{\partial x^2} - [CO_2(aq)][OH^-]k_{1f} + [HCO_3^-]k_{1r}$$

$$- [HCO_3^-][OH^-]k_{2f} + [CO_3^{2-}]k_{2r}$$

The diffusion coefficients for each species are taken from Gattrell et al.[2] and are listed in Table S2:

Table S2. Diffusion coefficients for reactive species

D_{CO_2}	$D_{HCO_3^-}$	$D_{CO_3^{2-}}$	D_{OH^-}
1.91×10^{-9}	9.23×10^{-10}	1.19×10^{-9}	5.27×10^{-9}

The boundary conditions of this equation in different electrolytes are briefly introduced below.

At time t > 0 and x = 0 (the interface of bulk solution and the boundary layer):

The electrolytes are x M K_2SO_4 + y M KX (X = Cl, Br, I, $2x + y = 1$) without buffering ability, thus the concentration of CO_2(aq), CO_3^{2-}, HCO_3^- and OH^- are similar;

At time t > 0 and x = δ (the electrolyte surface):

The current density determines the generation of OH^- on the electrode surface, while the current density and product distribution determine the consumption of CO_2. For the same halide ion, since K_2SO_4 was added as supporting electrolyte to maintain the concentration of cation ions, the current density did not change obviously with the change of the halide ion concentration at the same applied potential. Different product distributions will result in different CO_2 consumption on the electrode surface, however the difference in total carbon containing products is relatively small at the same applied potential. Thus, the boundary conditions of these equations are similar in the same halide ion electrolyte. Matlab was used to calculate the concentration distribution of various species

on the electrode surface, and the surface local pH was obtained. The local pH values are similar in different halide ions electrolyte under the same potential.

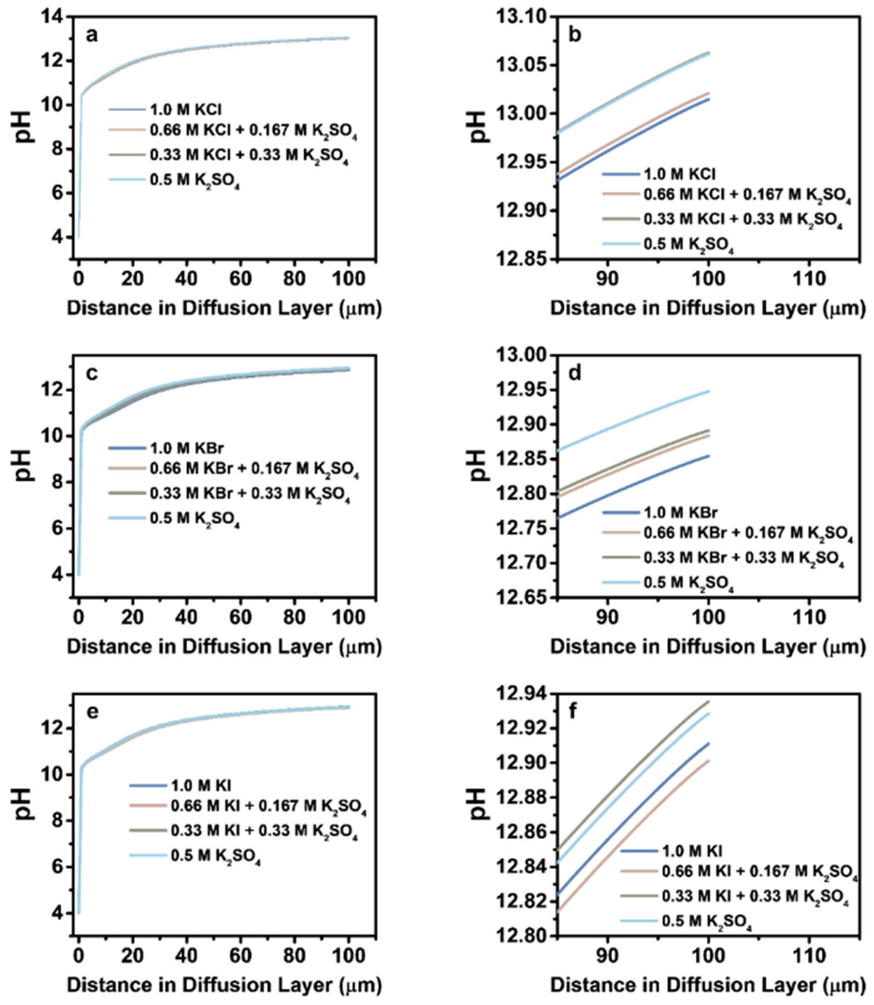

Figure S10. Simulated local pH profile as a function of distance in diffusion layer at −1.25 V vs RHE in (a) x M K_2SO_4 + y M KCl ($2x + y = 1$, $x = 0, 0.17, 0.33, 0.5$), (c) x M K_2SO_4 + y M KBr ($2x + y = 1$, $x = 0, 0.17, 0.33, 0.5$), (e) x M K_2SO_4 + y M KI ($2x + y = 1$, $x = 0, 0.17, 0.33, 0.5$) electrolytes after reaction for 20 seconds, where (b), (d) and (f) are the enlarged image of (a), (c) and (e) from 85 to 100 μm, respectively.

The effect of the cation concentration on the product distribution was investigated using aqueous $KHCO_3$ + K_2SO_4 mixed electrolyte. The C_{2+} FE reaches 71.7% with a current density of 73.4 mA/cm^2 in aqueous 0.1 M $KHCO_3$ + 0.2 M K_2SO_4 mixed electrolyte. After reaction in aqueous 0.1 M $KHCO_3$ + 0.2 M K_2SO_4 mixed electrolyte, catalysts were transferred into 0.1 M aqueous $KHCO_3$ electrolyte. The C_{2+} FE (70.2%) in 0.1 M aqueous $KHCO_3$ electrolyte is similar with that in 0.1 M $KHCO_3$ + 0.2 M K_2SO_4, while the current density is only about 40 mA/cm^2. The enhancement of C_{2+} activity is possibly caused by the increase of cation concentration. Thus, the concentration of aqueous electrolyte concentration needs to be kept constant during CO_2RR when studying the effect of specific adsorption of halide ions.

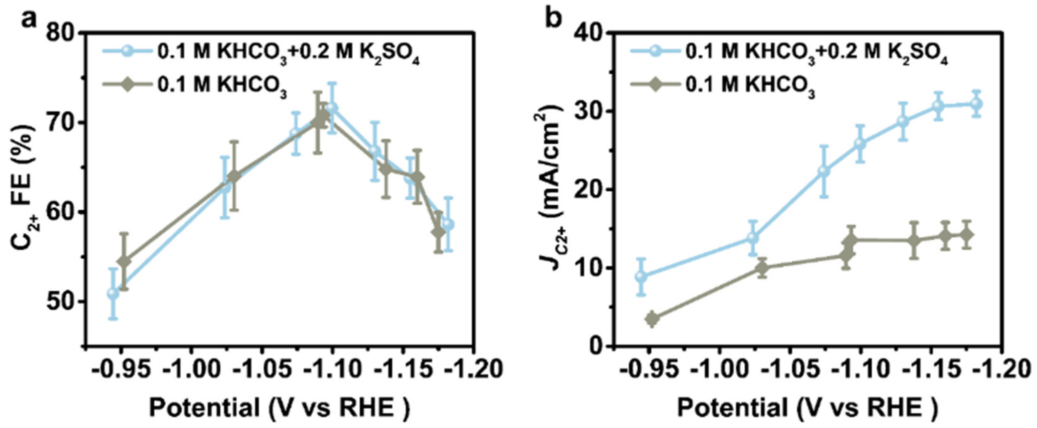

Figure S11. (a) C_{2+} FE and (b) C_{2+} partial current density in aqueous 0.1 M $KHCO_3$, 0.1 M $KHCO_3$ + 0.2 M K_2SO_4 mixed electrolyte.

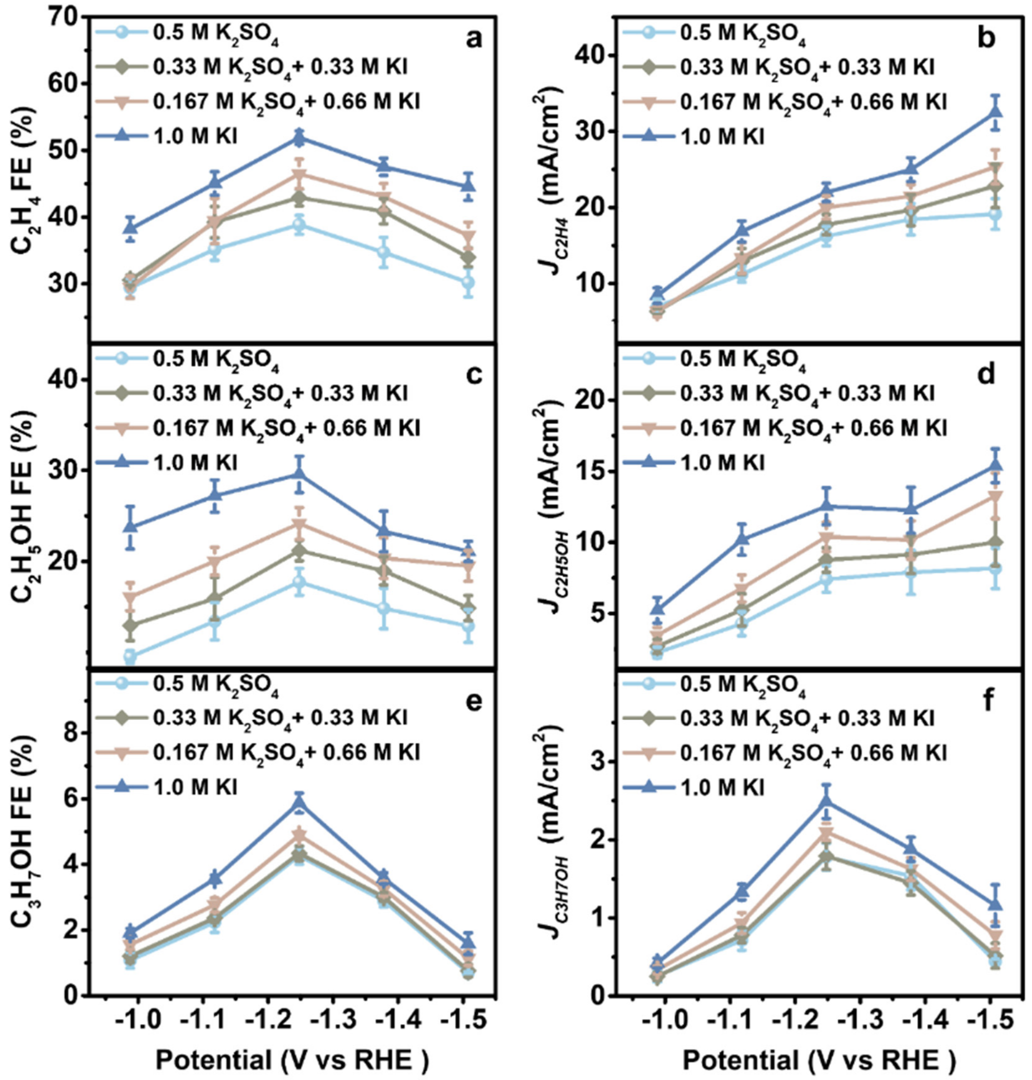

Figure S12. FE for (a) ethylene, (c) ethanol, (e) *n*-propanol and partial current density for (b) ethylene, (d) ethanol, (f) *n*-propanol reacted in aqueous KI and K_2SO_4 mixed electrolytes at various applied potentials.

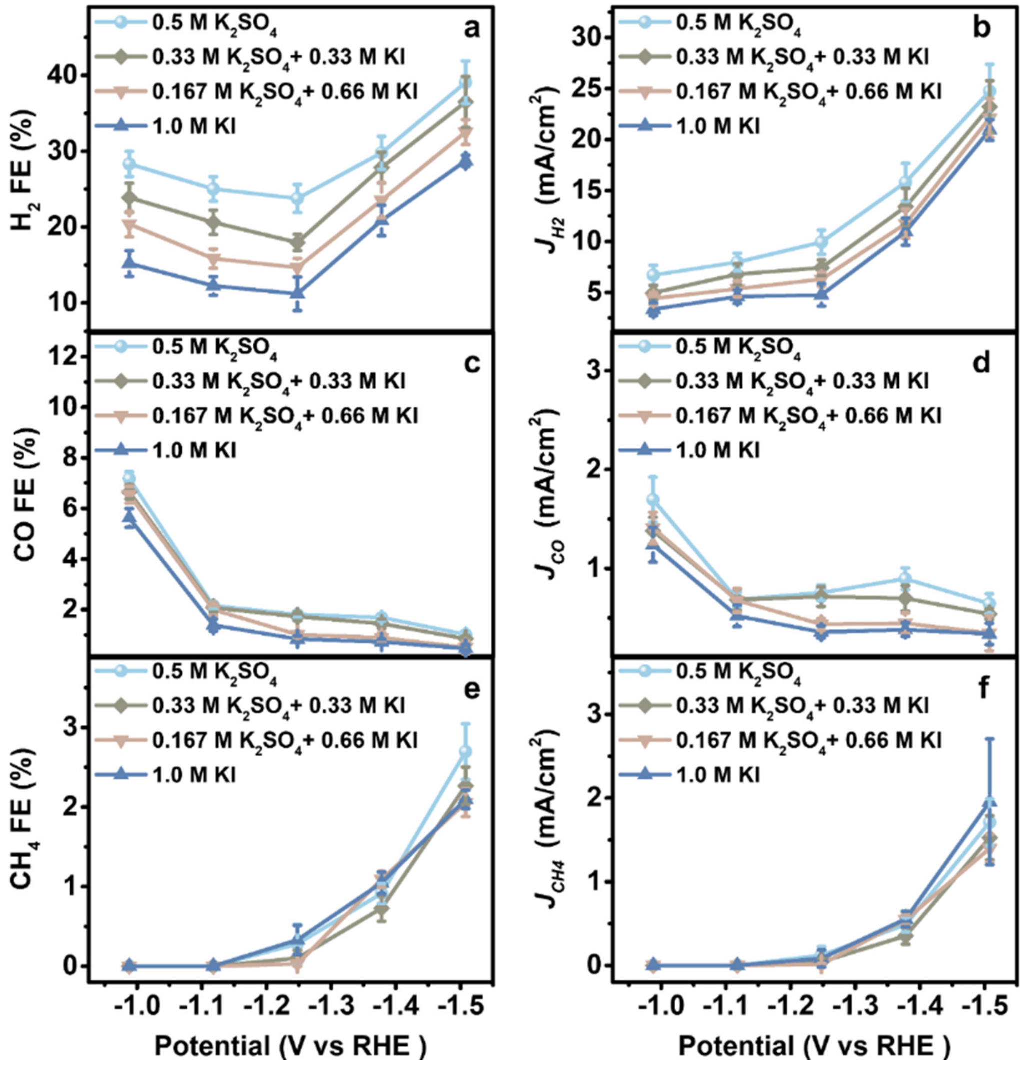

Figure S13. FE for (a) hydrogen, (c) carbon monoxide, (e) methane and partial current density for (b) hydrogen, (d) carbon monoxide, (f) methane reacted in aqueous KI and K_2SO_4 mixed electrolytes at various applied potentials.

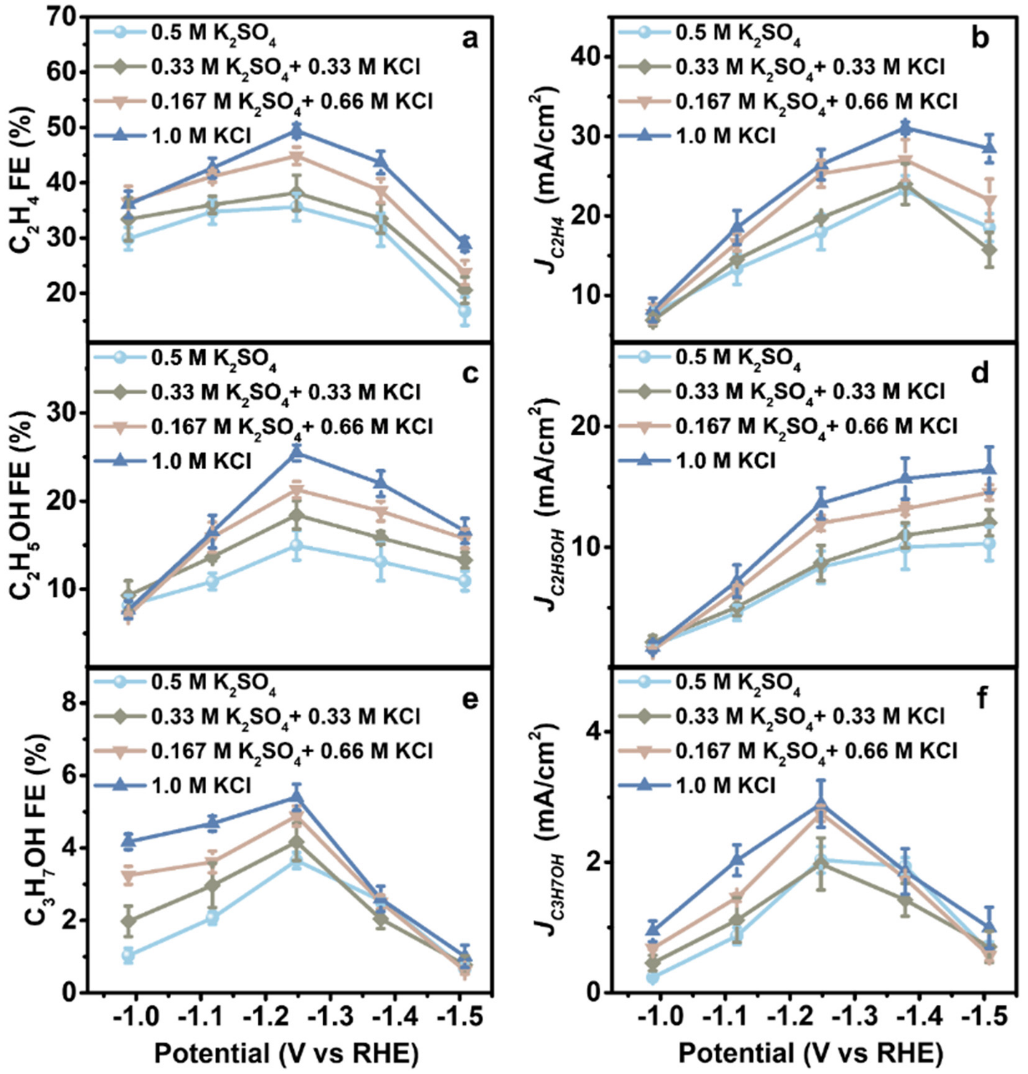

Figure S14. FE for (a) ethylene, (c) ethanol, (e) *n*-propanol and partial current density for (b) ethylene, (d) ethanol, (f) *n*-propanol reacted in aqueous KCl and K_2SO_4 mixed electrolytes at various applied potentials.

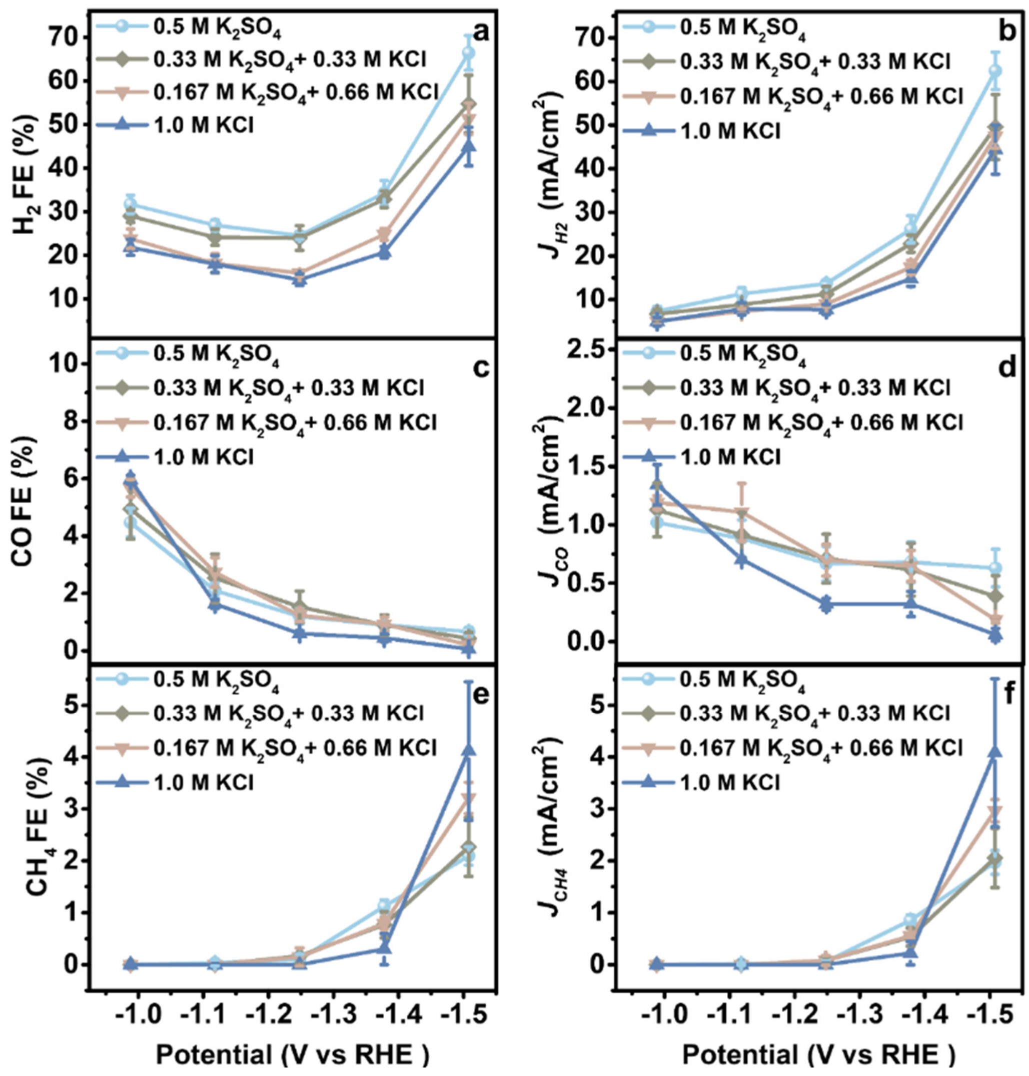

Figure S15. FE for (a) hydrogen, (c) carbon monoxide, (e) methane and partial current density for (b) hydrogen, (d) carbon monoxide, (f) methane reacted in aqueous KCl and K_2SO_4 mixed electrolytes at various applied potentials.

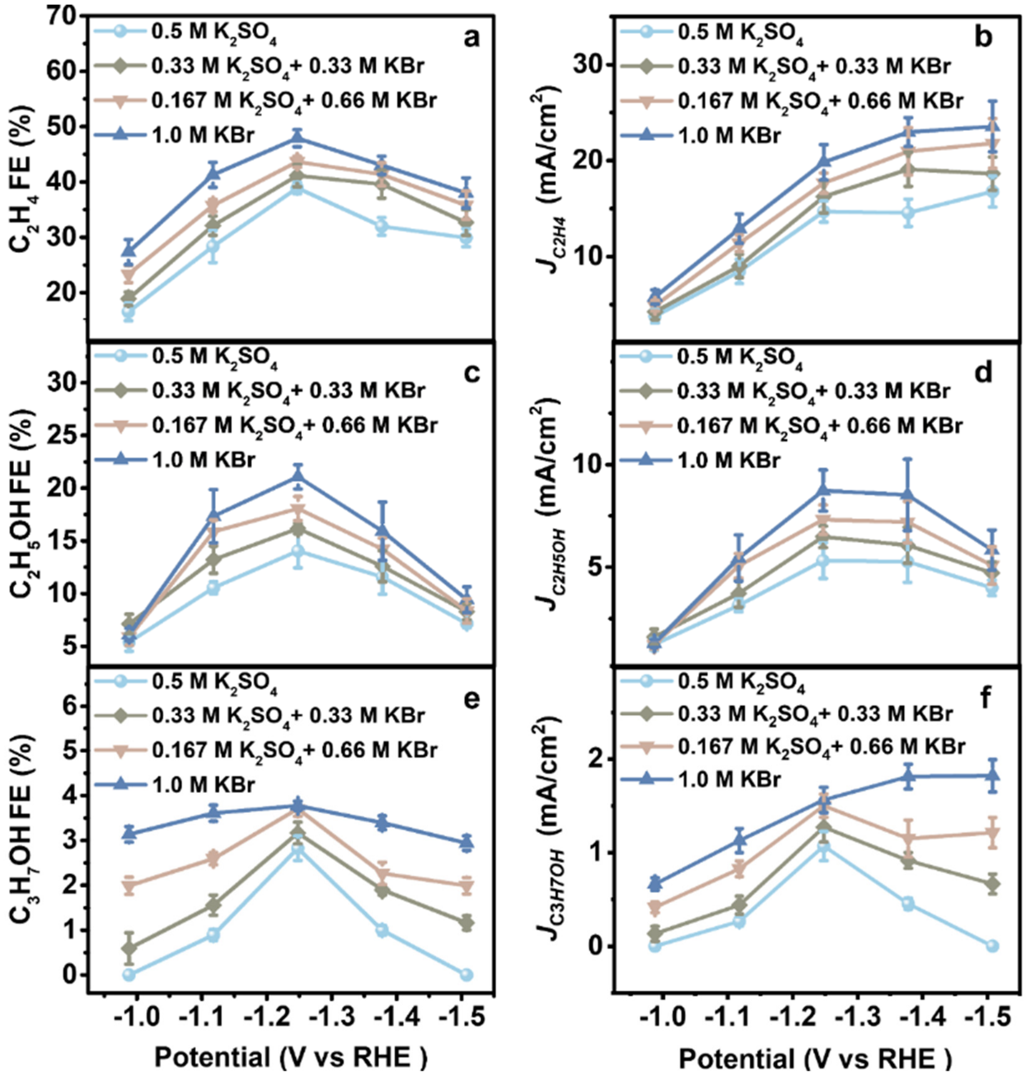

Figure S16. FE for (a) ethylene, (c) ethanol, (e) *n*-propanol and partial current density for (b) ethylene, (d) ethanol, (f) *n*-propanol reacted in aqueous KBr and K_2SO_4 mixed electrolytes at various applied potentials.

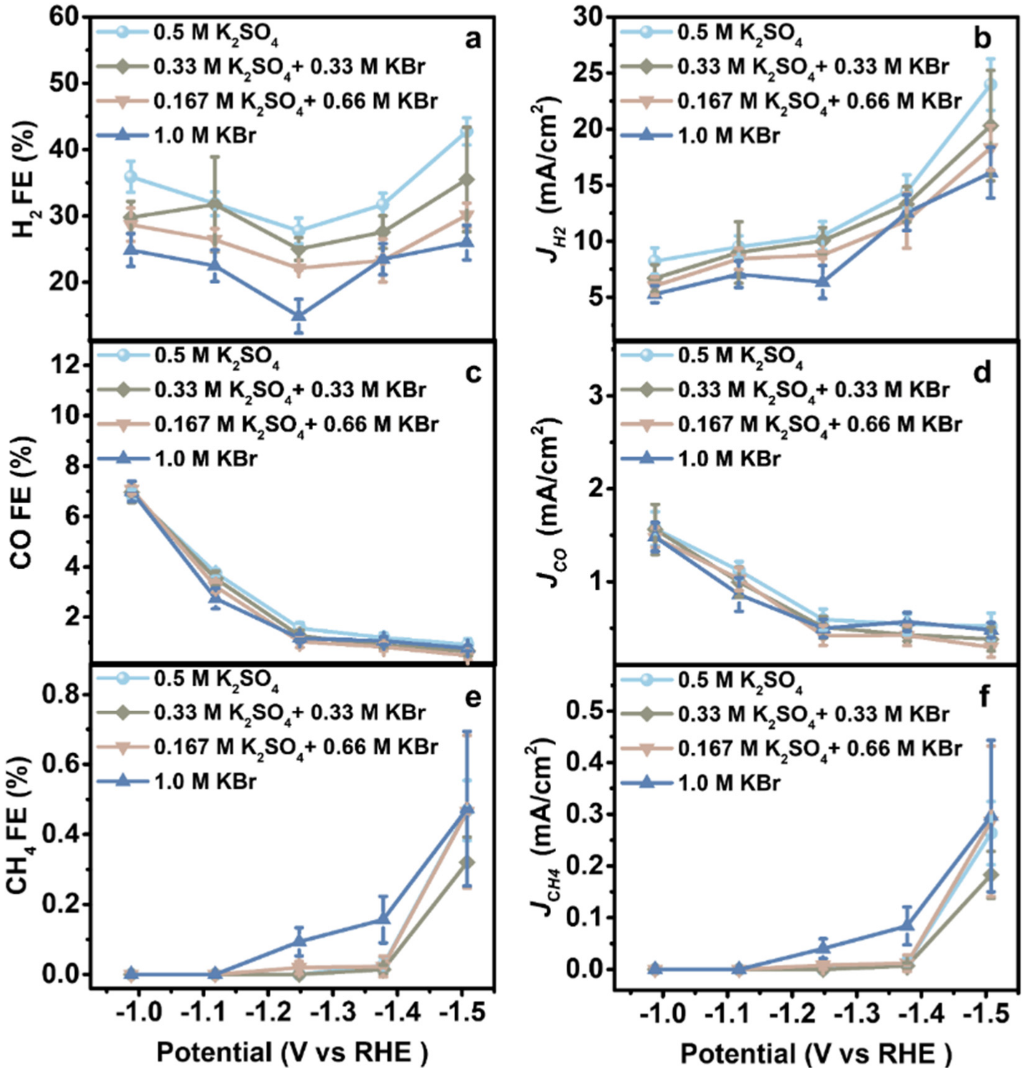

Figure S17. FE for (a) hydrogen, (c) carbon monoxide, (e) methane and partial current density for (b) hydrogen, (d) carbon monoxide, (f) methane reacted in aqueous KBr and K_2SO_4 mixed electrolytes at various applied potentials.

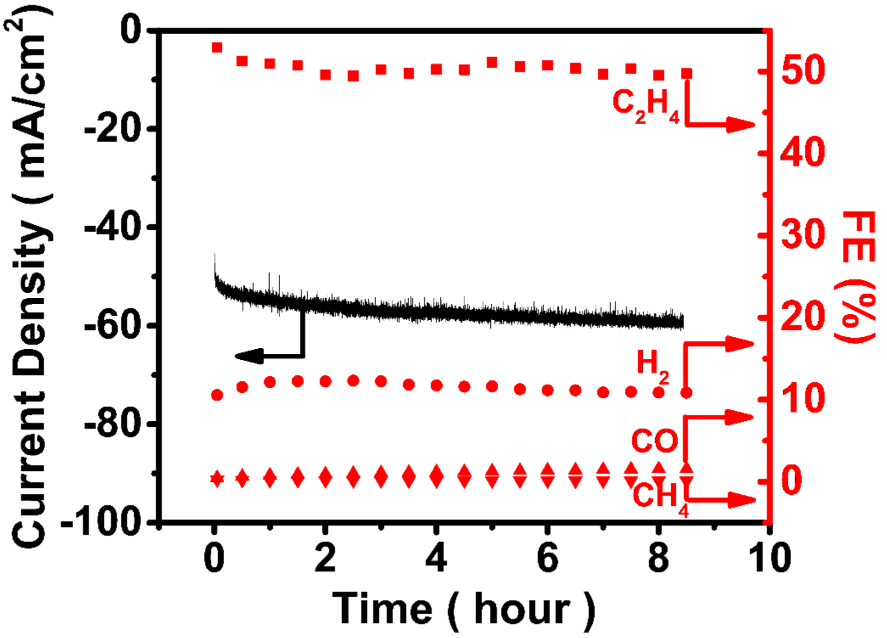

Figure S18. Stability of catalysts reaction in aqueous 1.0 M KI electrolyte.

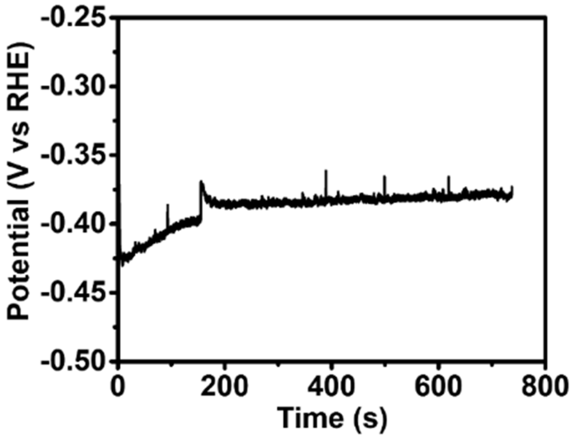

Figure S19. The applied potential when the current density is −1 mA/cm^2 during *in situ* Raman.

The reduction potential of CuI to Cu (E(CuI/Cu)) is also calculated.

$$\text{CuI(s)} + \text{e} \rightleftharpoons \text{Cu(s)} + \text{I}^{-}\text{(aq)} \quad E^{\ominus} = -0.1858\ \text{V}$$

Considering the concentration of I^{-} is 1 M, thus:

$$E(\text{CuI/Cu}) = E^{\ominus} - \frac{RT}{F}\ln\frac{c(\text{I}^{-})}{c^{\ominus}} = -0.1858\ \text{V}$$

The applied potential is −0.38 V vs RHE during *in situ* Raman, which is below −0.1858 V vs RHE. Thus, the CuI is reduced. Furthermore, the potential applied during CO_2RR is below −0.38 V vs RHE, therefore, CuI should have been reduced during CO_2RR.

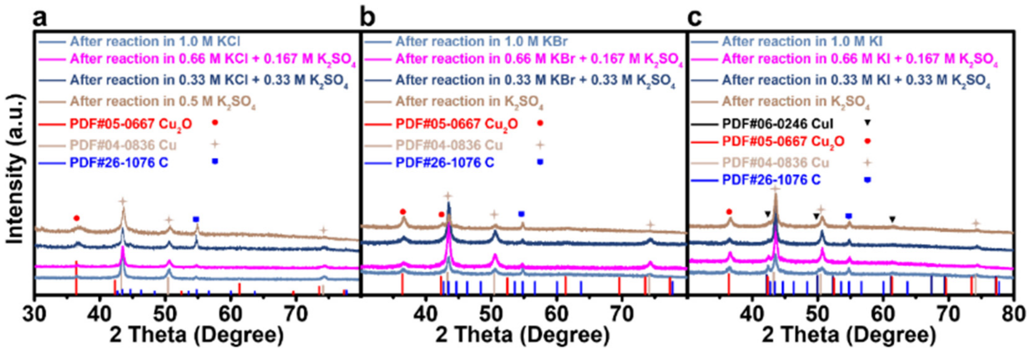

Figure S20. XRD patterns of catalysts acquired after pre-reduction at −1.25 V vs RHE in 0.2 M KX for 10 min and 1.0 M KX for 20 min, respectively, followed by reaction in various aqueous electrolytes with comparable reaction durations: (a-c) light blue: 30 min reaction in 1.0 M KCl. KBr and KI respectively; pink: 30 min reaction in 0.167 M K_2SO_4 + 0.66 M KCl, 0.167 M K_2SO_4 + 0.66 M KBr and 0.167 M K_2SO_4 + 0.66 M KI respectively; dark blue: 30 min reaction in 0.33 M K_2SO_4 + 0.33 M KCl, 0.33 M K_2SO_4 + 0.33 M KBr and 0.33 M K_2SO_4 + 0.33 M KI, respectively; brown: 30 min reaction in 0.5 M K_2SO_4.

The XRD characterization results show that the main species and crystal faces are Cu(111) and Cu(100). The ratio of the peak intensities of these two crystal planes (Table S3) show that Cu(111)/Cu(100) is around 3 after reaction in electrolytes containing halide ions. Specifically, Cu(111)/Cu(100) is around 2.9 after reaction in x M K_2SO_4 + y M KCl ($2x + y = 1$, $x = 0$, 0.17, 0.33, 0.5) electrolytes; while Cu(111)/Cu(100) is around 3.2 when reacting in x M K_2SO_4 + y M KBr ($2x + y = 1$, $x = 0$, 0.17, 0.33, 0.5) electrolytes; moreover, Cu(111)/Cu(100) is around 3.0 after reaction in x M K_2SO_4 + y M KI ($2x + y = 1$, x = 0, 0.17, 0.33, 0.5) electrolytes. Thus, Cu(111)/Cu(100) are similar after reaction in electrolyte containing the same halide ion.

Table S3. The ratio of Cu(111)/Cu(100) after reaction in different electrolytes.

Electrolyte (pre-reduction in KCl)	Cu(111)/ Cu(100)	Electrolyte (pre-reduction in KBr)	Cu(111)/ Cu(100)	Electrolyte (pre-reduction in KI)	Cu(111)/ Cu(100)
1.0 M KCl	2.99	1.0 M KBr	3.13	1.0 M KI	2.96
0.66 M KCl + 0.167 M K_2SO_4	2.87	0.66 M KBr + 0.167 M K_2SO_4	3.23	0.66 M KI + 0.167 M K_2SO_4	2.98
0.33 M KCl+ 0.33 M K_2SO_4	2.97	0.33 M KBr+ 0.33 M K_2SO_4	3.19	0.33 M KI+ 0.33 M K_2SO_4	3.08
0.5 M K_2SO_4	2.93	0.5 M K_2SO_4	3.18	0.5 M K_2SO_4	3.09

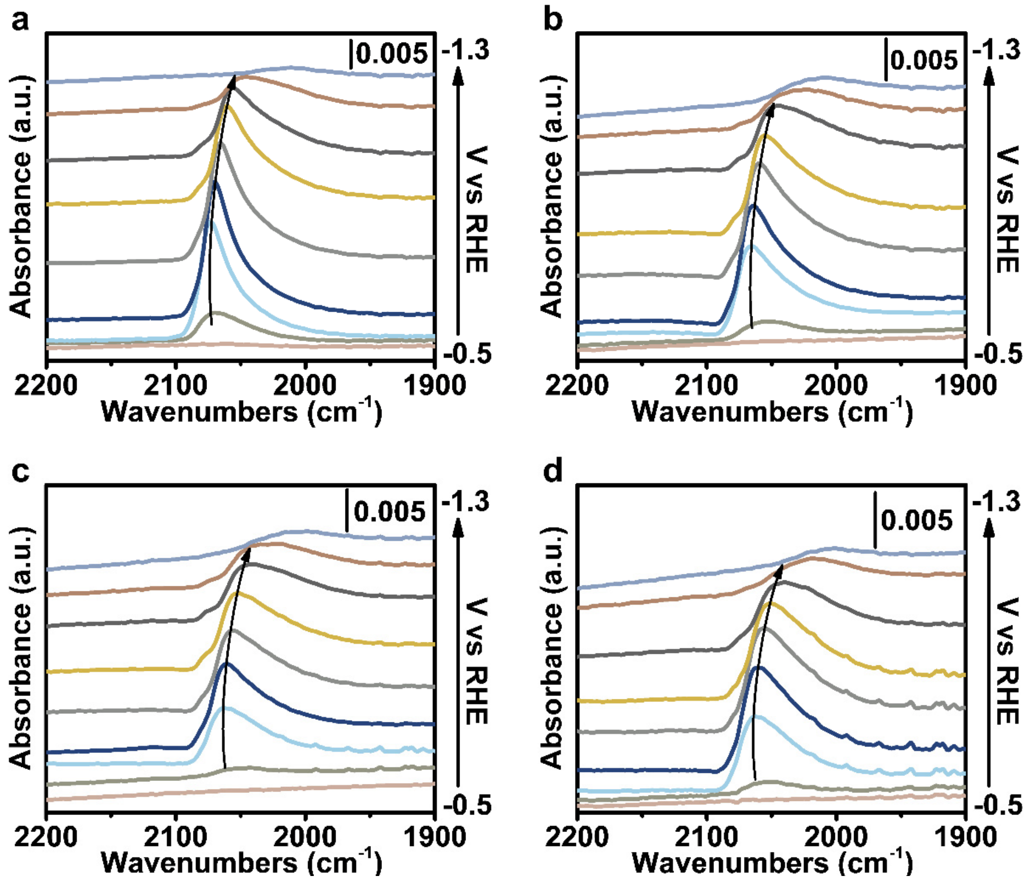

Figure S21. *In situ* ATR-SEIRAS spectra obtained in aqueous (a) 0.5 M K_2SO_4, (b) 0.33 M K_2SO_4 + 0.33 M KBr, (c) 0.167 M K_2SO_4 + 0.66 M KBr and (d) 1.0 M KBr electrolytes with catalysts loading on Si wafer covered by Au. The catalysts were pre-reduced in aqueous 0.2 M KBr for 10 min, 1.0 M KBr for 20 min, respectively, at −1.25 V vs RHE.

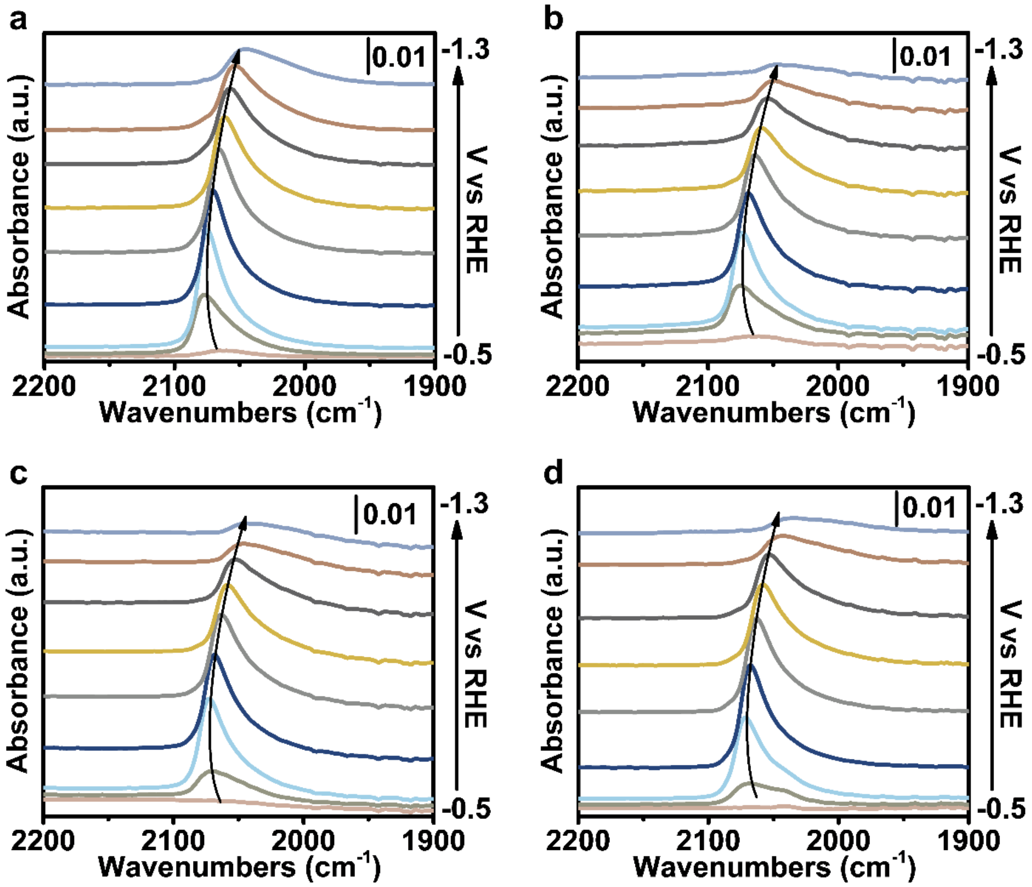

Figure S22. *In situ* ATR-SEIRAS spectra obtained in aqueous (a) 0.5 M K_2SO_4, (b) 0.33 M K_2SO_4 + 0.33 M KCl, (c) 0.167 M K_2SO_4 + 0.66 M KCl and (d) 1.0 M KCl electrolytes with catalysts loading on Si wafer covered by Au. The catalysts were pre-reduced in aqueous 0.2 M KCl for 10 min, 1.0 M KCl for 20 min, respectively, at −1.25 V vs RHE.

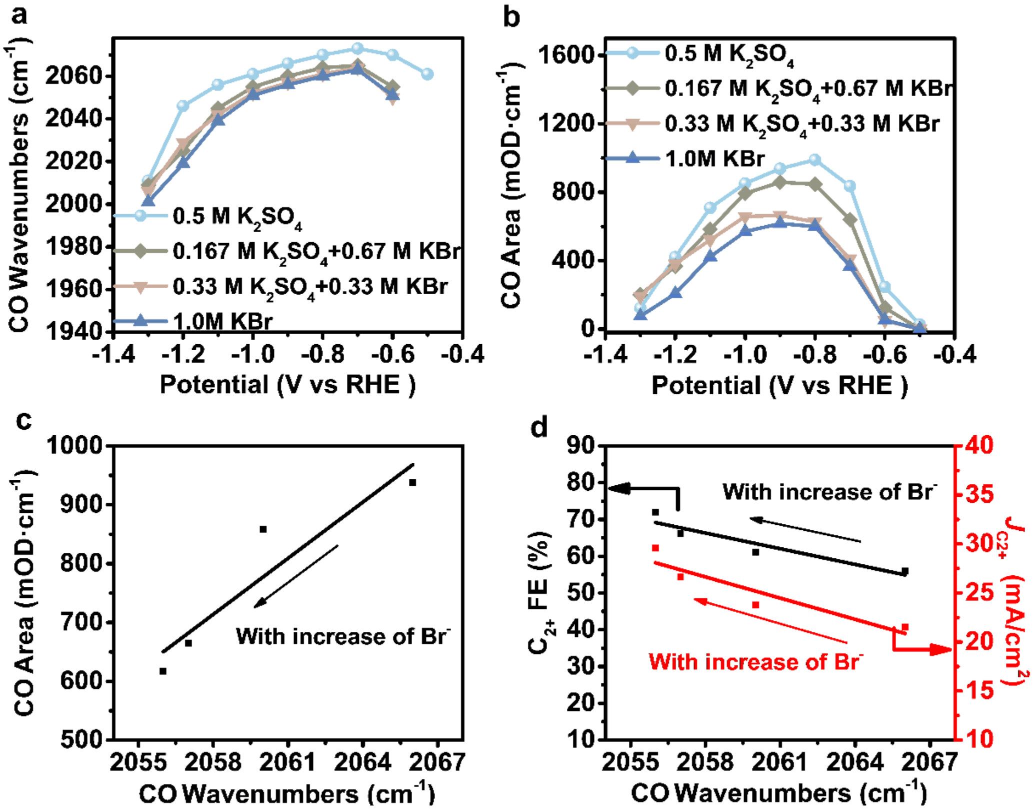

Figure S23. (a) CO wavenumbers and (b) CO areas vary with potential in different aqueous electrolytes. (c) Variation of CO areas with CO wavenumbers at −0.9 V vs RHE as the Br^- concentration varies, (d) Variation of C_{2+} FE and C_{2+} partial current density at −1.25 V vs RHE with CO wavenumbers at −0.9 V vs RHE as the Br^- concentration varies. The catalysts were pre-reduced in aqueous 0.2 M KBr for 10 min, 1.0 M KBr for 20 min, respectively, at −1.25 V vs RHE.

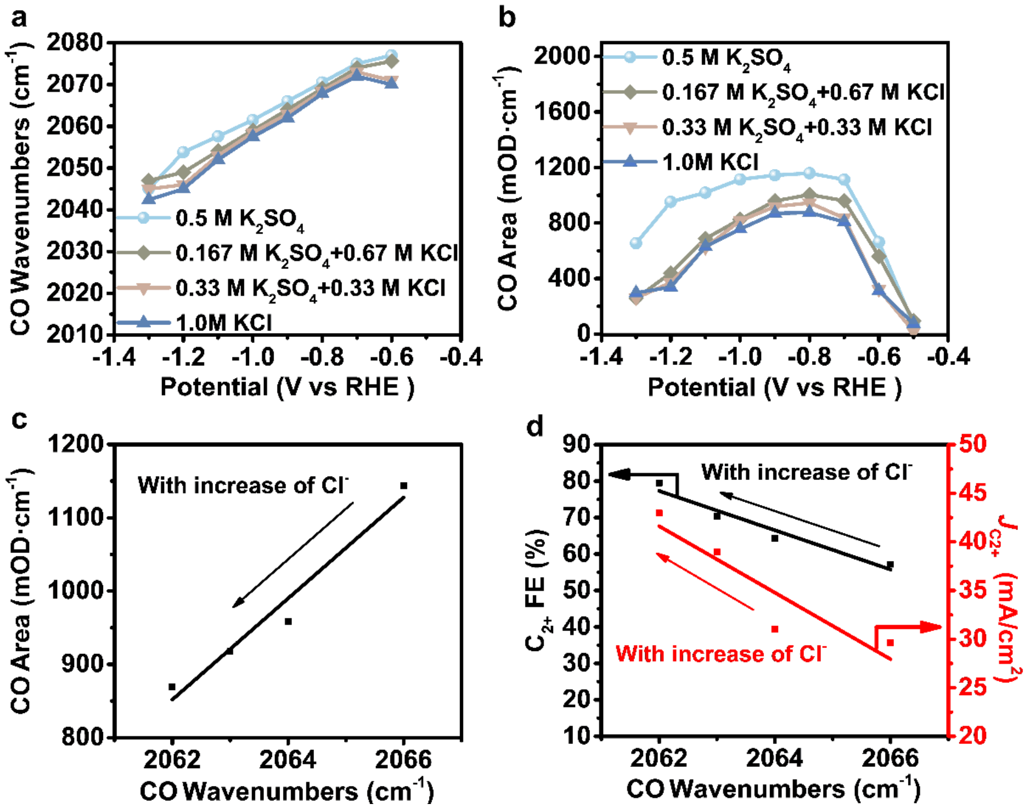

Figure S24. (a) CO wavenumbers and (b) CO areas vary with potential in different aqueous electrolytes. (c) Variation of CO areas with CO wavenumbers at −0.9 V vs RHE as the Cl^- concentration varies, (d) Variation of C_{2+} FE and C_{2+} partial current density at −1.25 V vs RHE with CO wavenumbers at −0.9 V vs RHE as the Cl^- concentration varies. The catalysts were pre-reduced in aqueous 0.2 M KCl for 10 min, 1.0 M KCl for 20 min, respectively, at −1.25 V vs RHE.

References

1. W. Deng, L. Zhang, L. Li, S. Chen, C. Hu, Z. J. Zhao, T. Wang and J. Gong, *J. Am. Chem. Soc.*, 2019, **141**, 2911-2915.
2. N. Gupta, M. Gattrell and B. MacDougall, *J. Appl. Electrochem.*, 2005, **36**, 161-172.
